# Tiam1/Rac1 complex controls *Il17a* transcription and autoimmunity

**DOI:** 10.1038/ncomms13048

**Published:** 2016-10-11

**Authors:** Ahmed T. Kurdi, Ribal Bassil, Marta Olah, Chuan Wu, Sheng Xiao, Mariko Taga, Michael Frangieh, Thomas Buttrick, William Orent, Elizabeth M. Bradshaw, Samia J. Khoury, Wassim Elyaman

**Affiliations:** 1 Ann Romney Center for Neurologic Diseases, Brigham and Women's Hospital and Harvard Medical School, Boston, Massachusetts 02115, USA; 2 Program in Translational NeuroPsychiatric Genomics, Department of Neurology, Brigham and Women's Hospital, Harvard Medical School, Broad Institute at Harvard University and MIT, Boston, Massachusetts 02115, USA; 3 Abu Haidar Neuroscience Institute, American University of Beirut Medical Center, Riad El Solh, Beirut 1107 2020, Lebanon

## Abstract

RORγt is a master transcription factor of Th17 cells and considered as a promising drug target for the treatment of autoimmune diseases. Here, we show the guanine nucleotide exchange factor, Tiam1, and its cognate Rho-family G protein, Rac1, regulate interleukin (IL)17A transcription and autoimmunity. Whereas *Tiam1* genetic deficiency weakens IL-17A expression partially and inhibits the development of experimental autoimmune encephalomyelitis (EAE), deletion of *Rac1* in T cells exhibits more robust effects on Th17 cells and EAE. We demonstrate Tiam1 and Rac1 form a complex with RORγt in the nuclear compartment of Th17 cells, and together bind and activate the *Il17* promoter. The clinical relevance of these findings is emphasized by pharmacological targeting of Rac1 that suppresses both murine and human Th17 cells as well as EAE. Thus, our findings highlight a regulatory pathway of Tiam1/Rac1 in Th17 cells and suggest that it may be a therapeutic target in multiple sclerosis.

## Abstract (web summary)

Tiam1 is a guanine nucleotide exchange factor for the Rho-family GTPase Rac1. Here, the authors show that nuclear Tiam1 and Rac1 bind to RORγt on the IL-17 promoter, activating its transcription, and that inhibiting Tiam1/Rac1 is beneficial in a mouse model of autoimmunity.

## Graphical abstract

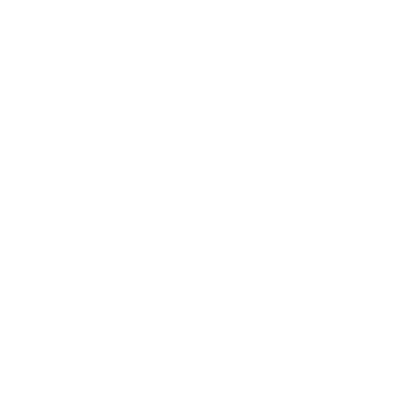

T helper (Th)17 cells are considered to play a pivotal role in the pathogenesis of multiple sclerosis (MS) as well as its animal model, experimental autoimmune encephalomyelitis (EAE)^1^,^2^. Naive CD4^+^ T cells differentiate into Th17 cells when activated in the presence of transforming growth factor (TGF)-β and interleukin (IL)-6 (ref. 3). Alongside their signature cytokines, IL-17A and IL-17F, Th17 cells are characterized by their expression of pro-inflammatory cytokines such as IL-22 and granulocyte–macrophage colony-stimulating factor (GM-CSF)^4^,^5^. The pro-inflammatory function of IL-17A is demonstrated by the fact that IL-17A deficient mice were protected from EAE^6^. IL-17A neutralization is a promising therapy for Th17-associated autoimmune diseases such as psoriasis, ankylosing spondylitis and MS^7^,^8^,^9^. Recent success in clinical trials for the treatment of psoriasis and rheumatoid arthritis with biologics that inhibit the IL17A-IL17R axis (Ixekizumab and Brodalumab) further underscores the importance of this pathway in human autoimmunity^10^,^11^,^12^.

The transcription factor RAR-related orphan receptor gamma (RORγt), recognized as the master transcription factor of Th17 cells, promotes Th17 cell differentiation and is essential for the development of murine and human Th17 cells^13^,^14^. RORγt deficient mice are resistant to autoimmune diseases^13^. RORγt functions in concert with IL-6/STAT3, TGFβ1, and IL-23 to drive the generation of pathogenic Th17 cells^15^,^16^,^17^. RORγt also belongs to the nuclear hormone receptors (NHRs), a well characterized family of transcription factors composed of modular protein structures comprising DNA- and ligand-binding domains (DBDs and LBDs). While DBDs confer gene target site specificity, LBDs act as control switches for NHR function^18^. The RORγt LBD is therefore an ideal domain that can be targeted via small molecules. Numerous studies have identified the downstream genomic targets of RORγt in CD4^+^ T cells^19^,^20^,^21^, however, very little is known about endogenous ligands that control RORγt function in Th17 cells.

Rho-GTPases such as Rac1 function as molecular switches that cycle between active GTP-bound and inactive GDP-bound states. In their active state, they interact with effector molecules and stimulate signalling pathways controlling cytoskeletal dynamics, membrane trafficking and gene expression programs^22^,^23^. As a well characterized membrane-bound signal transducing molecule, Rac1 is involved in regulating cell motility and adhesion in addition to the progression of the cell cycle, mitosis, cell death and gene expression^24^. Since an elevated level of expression and activity of this protein has been associated with cancer metastasis, direct regulation of Rac1 activity is a potential strategy employed in the treatment of certain cancers^25^. Rac1 regulates several signalling pathways in cancer cells including the Wnt/β-catenin pathway by stimulating the assembly of β-catenin-lymphoid enhancer factor-1 complex^26^. T lymphoma invasion and metastasis 1 (Tiam1) is a guanine nucleotide exchange factor (GEF) of Rac1 that is believed to act as an oncogene^27^. Acting principally upstream of Rac1, Tiam1 is mainly involved in the regulation of Rac1-mediated signalling pathways including cytoskeletal activities, endocytosis and membrane trafficking as well as cell polarity, migration, adhesion, carcinogenesis and metastasis^28^,^29^. Together, the Tiam1/Rac1 complex constitutes a critical component in the biology of human tumours, in both transformed cells and the accessory cells of the tumour microenvironment^30^,^31^.

In the present study, we investigate the role of Tiam1/Rac1 signalling in mediating murine and human Th17 cell development and altering cytokine expression profile. Using genetic mouse models as well as small molecule inhibitors, we identify a novel role of the Tiam1/Rac1 complex in the regulation of RORγt-mediated *Il17a* transcription and autoimmune inflammation.

## Results

### Increased expression of Tiam1 and Rac1 in Th17 cells

**Figure 1:**
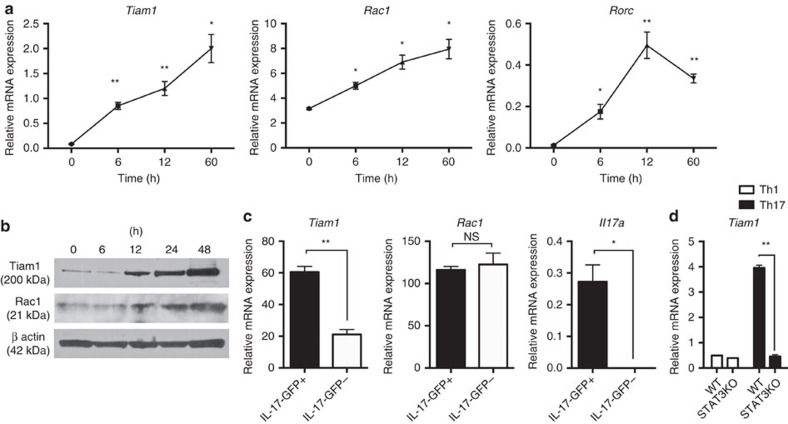
Increased expression of Tiam1 and Rac1 in murine Th17 cells. (**a**) Temporal Tiam1 and Rac1 gene expression in mouse Th17 cells. Naive CD4^+^ T cells were cultured under Th17 cell polarizing conditions with recombinant IL-6 plus TGF-β1 for 0–60 h (h) and *Tiam1* and *Rac1* mRNA expression was assessed by Taqman PCR. Control *Rorc* expression is shown. (**b**) Immunoblots of Tiam1 and Rac1 protein expression in naive CD4^+^ polarized under Th17 conditions for the indicated time points (0–48 h). β-actin was used as internal control for protein loading. (**c**) Relative mRNA expression of Tiam1 and Rac1 in CD4^+^ T cells isolated from MOG_35–55_-immunized IL-17A-GFP knock-in mice. IL-17A-GFP^+^ and IL-17A-GFP^−^ cells were separated by flow cell sorting from spleens of EAE mice 10 days after immunization with MOG_35–55_ peptide and *Tiam1* and *Rac1* mRNA expression was analyzed by quantitative Taqman PCR. Control *Il17a* expression is shown. (**d**) STAT3 genetic deficiency suppressed the induction of Tiam1 expression in Th17 cells. CD4^+^ T cells were isolated from spleens of naïve STAT3KO and WT mice, T cells were differentiated under Th1 and Th17 cells for 24 h and *Tiam1* mRNA expression was assessed by Taqman PCR. Data represent mean±s.e.m. of a representative experiment each performed in triplicate. *P<0.05; **P<0.01 by Student *t*-test. Data are representative of 2–3 biological replicates with similar results.

We investigated a possible role of the Tiam1/Rac1 complex in Th17 cells. We found that in Th17 cells, Tiam1 expression is induced within 6 h after polarizing naive CD4^+^CD62L^hi^CD44^low^ cells with TGF-β1 and IL-6 as measured at the gene and protein levels (Fig. 1a,b; Supplementary Fig. 1). Moreover, Rac1 expression was detected in naive CD4^+^ T cells and was further increased at both the mRNA and protein levels (Fig. 1a,b). To ascertain the specificity of high Tiam1 expression among Th17 cells, we investigated the relative expression of Tiam1 in CD4^+^ T cells isolated from myelin oligodendrocyte glycoprotein peptide (MOG)_35–55_-immunized IL-17A-GFP knock-in mice 10 days after immunization. IL-17A-GFP^+^ and IL-17A-GFP^−^ cells were separated by fluorescence-activated cell sorting (FACS) and Tiam1 expression was analyzed by quantitative PCR. Tiam1 was increased nearly by threefolds in IL-17A-GFP^+^ lymphocytes compared with the IL-17A-GFP^−^ counterpart, while Rac1 was constitutively expressed at the gene level in both cell populations (Fig. 1c). Thus, the guanine nucleotide exchange factor, Tiam1, is specifically expressed in IL-17A-producing CD4^+^ T cells. Given the rapid induction of Tiam1 in Th17 cells, we sought to investigate molecular signalling pathways that are involved in Tiam1 transcription. STAT3, a key downstream mediator of IL-6 signalling, binds the IL-17 promoter and induces RORγt production^32^. To study the potential role of STAT3 in inducing Tiam1 expression, we extracted high-resolution transcription factor-DNA interaction profiles from chromatin immunoprecipitation and high-throughput sequencing (ChIP-seq) data deposited on Gene Expression Omnibus (GEO)^32^. STAT3 demonstrated high-confidence binding to the promoter region of all the Tiam1 isoforms (Supplementary Fig. 2). To study whether this interaction is functional, we took advantage of the STAT3 conditional knockout mouse model. Naïve CD4^+^ T cells from *CD4-cre x STAT3*^fl/fl^ mice and *STAT3*^fl/fl^ littermates were differentiated under Th17 polarizing conditions and Tiam1 expression was assessed 24 h after *in vitro* stimulation. We found that STAT3 deficient Th17 cells expressed significantly lower Tiam1 levels than their wild-type (WT) counterpart (Fig. 1d).

### Tiam1 deficiency partially reduces IL-17A expression and EAE

**Figure 2:**
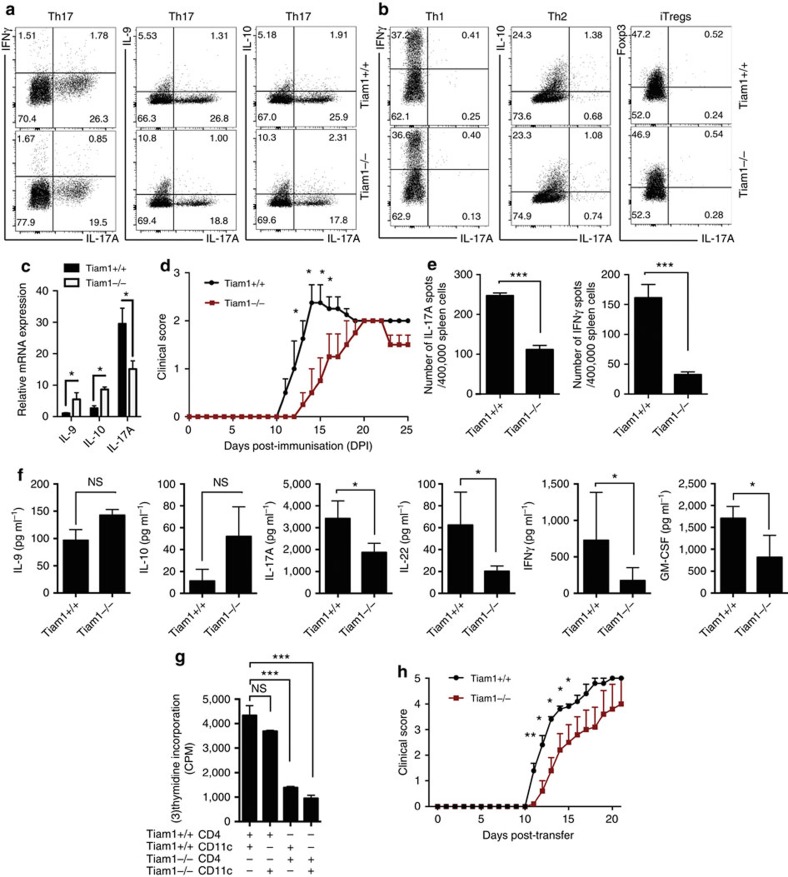
Modest effects of Tiam1 genetic deficiency on IL-17A expression and EAE. (**a**) Naive CD4^+^ T cells were isolated from Tiam1 deficient mice (*Tiam1−*/−) or control (*Tiam1+*/+) littermates and were stimulated *in vitro* under Th17 cell polarizing conditions and cytokine profile was analyzed by intracellular cytokine staining. (**b**) Flow cytometry cytokine expression of *Tiam1−*/− and control *Tiam1+*/+ CD4^+^ T cells differentiated under Th1, Th2 and iTregs. (**c**) Analysis of cytokine expression by Taqman PCR in *Tiam1−*/− Th17 cells 4 days after cell differentiation. (**d**) Tiam1 genetic deficiency ameliorates clinical EAE clinical score. EAE was induced in *Tiam1−*/− and control *Tiam1+*/+ littermates with MOG_35–55_/CFA and mice were monitored for clinical EAE symptoms for up to 25 days post-immunization (DPI). Mean maximal EAE score (±s.e.m.) of *Tiam1−*/− was compared with that of control *Tiam1+*/+ mice by two-tailed Mann–Whitney *U* test. Statistical differences (*P<0.05) between the two groups on day 13–16 are shown (*n*=12–15 mice per group). (**e**) ELISPOT data of the frequency of IL-17A and IFNγ-producing cells in MOG_35–55_-activated spleen cells of immunized *Tiam1−*/− and *Tiam1+*/+ mice on day 8–10 after immunization (*n*=3–5 mice per group). (**f**) Cytokine expression of MOG_35–55_-activated spleen cells analyzed by Luminex assay. Results are representative of three independent experiments with three mice per group. (**g**) On day 10 post-immunization, splenocytes of *Tiam1−*/− and *Tiam1+*/+ mice were harvested, CD4^+^ T cells and CD11c^+^ dendritic cells were criss-crossed (DC:CD4 1:4 ratio) and tested for MOG_35–55_-specific proliferation. Data are shown as mean±s.e.m. *P<0.05; **P<0.01; ***P<0.001 by Student *t*-test. *NS*, non-significant. (**h**) Mean EAE scores (±s.e.m.) after adoptive transfer of MOG_35–55_-restimulated WT or *Tiam1−*/− CD4^+^ T cells. *Tiam1−*/− and *Tiam1+*/+ mice were immunized, LN cells were isolated and re-activated with MOG_35–55_ peptide (20 μg) in the presence of WT DCs and IL-23 for 2 days, and 0.75 million CD4^+^ T cells were injected into Rag1 deficient mice. The mean maximal score of recipients of *Tiam1−*/− compared with control *Tiam1+*/+ cells was compared by two-tailed Mann–Whitney *U* test. Statistical differences (*P<0.05) between the two groups on day 11–15 are shown (*n*=5 mice per group, representative of two independent experiments of total 10 mice per group).

Given the induction of Tiam1/Rac1 in Th17 cells, we investigated the role of the GEF, Tiam1, in IL-17 expression. Naive CD4^+^ T cells purified from splenocytes of *Tiam1−*/− mice and their control WT littermates (*Tiam1+*/+) were differentiated for 4 days under Th1, Th2, Th17 and iTreg conditions and then analyzed by intracellular cytokine staining. We found that genetic deletion of Tiam1 moderately reduced IL-17A production and increased IL-9 and IL-10, while IFNγ expression was not altered in Th17 cells (Fig. 2a). This phenomenon was specific to Th17 cells since cytokine profile of CD4^+^ T cells polarized *in vitro* under Th1, Th2 and iTreg conditions were not influenced by Tiam1 genetic deletion (Fig. 2b). The modest change in cytokine expression in Th17 cells was also reflected at the gene level with a twofold reduction in *Il17a* production and a notable increase in *Il9* and *Il10* expression in *Tiam1−*/− cells (Fig. 2c).

Since Tiam1 appears to contribute moderately to the IL-17A-producing capacity of Th17 cells, we were interested in investigating how this translates *in vivo* at the disease level. EAE was induced in *Tiam1−*/− and WT *Tiam1+*/+ control mice by immunization with MOG_35–55_ in complete Freund adjuvant (CFA) that was administered subcutaneously and followed by intraperitoneal (i.p.) injection of pertussis toxin (PT). We found that EAE induction was delayed in the *Tiam1−*/− mice compared with the *Tiam1+*/+ littermates and the disease course was milder (Fig. 2d). This delay is statistically significant particularly when analyzed from disease onset until 15 days post-immunization (*P*<0.0015 by two-tailed Mann–Whitney *U* test) (Fig. 2d). Analysis of the cytokine profile of MOG_35–55_-reactive CD4^+^ T cells was carried out by Enzyme-Linked ImmunoSpot (ELISPOT) technique as well as by Luminex bead-based assay. Accordingly, spleen cells were isolated from immunized mice 8–10 days after immunization and cells were exposed to MOG_35–55_ peptides (10 μg ml^−1^) for 36 h. We found that splenocytes from *Tiam1−*/− mice produced significantly less IL-17A and IFNγ compared with *Tiam1+*/+ control mice (Fig. 2e). This was confirmed by Luminex assay where the release of IL-17A and IFNγ in the culture supernatants was lower in MOG_35–55_-reactive T cells from *Tiam1−*/− mice. We also observed a decrease in IL-22 and GM-CSF, while IL-9 and IL-10 were slightly but not significantly upregulated in the absence of Tiam1 (Fig. 2f). Given that *Tiam1−*/− mice are a germline knockout mouse model that doesn't distinguish between the effects of Tiam1 deficiency in T cells from its deficiency in antigen-presenting cells (APCs), it is possible that the observed decrease in IFNγ production could be linked to both T-cell-dependent and independent mechanisms.

To measure the influence of Tiam1 on the magnitude of myelin-specific T-cell response, we analyzed MOG_35–55_-induced spleen cell proliferation in a criss-cross experiment. Thus, we isolated CD4^+^ T cells and CD11c^+^ dendritic cells from spleens of *Tiam1−*/− and *Tiam1+*/+ mice 10 days after immunization and cells were activated with MOG_35–55_ peptide for two days followed by quantitation of cell proliferation using [^3^H]*-*thymidine incorporation method. We found that although Tiam1 deficiency in CD11c^+^ cells reduced slightly but not significantly their antigen-presenting function, lack of Tiam1 in CD4^+^ T cells had marked effects on their proliferation (Fig. 2g). To establish a more direct comparison of the encephalitogenicity of *Tiam1−*/− versus WT donor T cells, we utilized an adoptive transfer EAE model. Draining lymph nodes (LNs) from MOG_35–55_/CFA-immunized *Tiam1−*/− and *Tiam1+*/+ were re-activated *in vitro* with MOG_35–55_ peptide plus recombinant IL-23 in the presence of WT dendritic cells according to established protocols for 2 days^33^ followed by transfer of equal numbers of CD4^+^ T cells replace in with to Rag1*−*/− lymphopenic mice that were monitored for clinical disease development. We found that EAE severity was slightly but significantly reduced in recipients of Tiam1-deficient T cells compared with WT T cells (mean maximal score for *Tiam1−*/− T cells compared with WT T cells between days 11 and 15 post-transfer; *P*<0.05 by Mann–Whitney *U* test; *n*=5/group) (Fig. 2h) leaving room for exploration of the downstream GTPase, Rac1.

### Rac1 is required for IL-17A expression and induction of EAE

**Figure 3:**
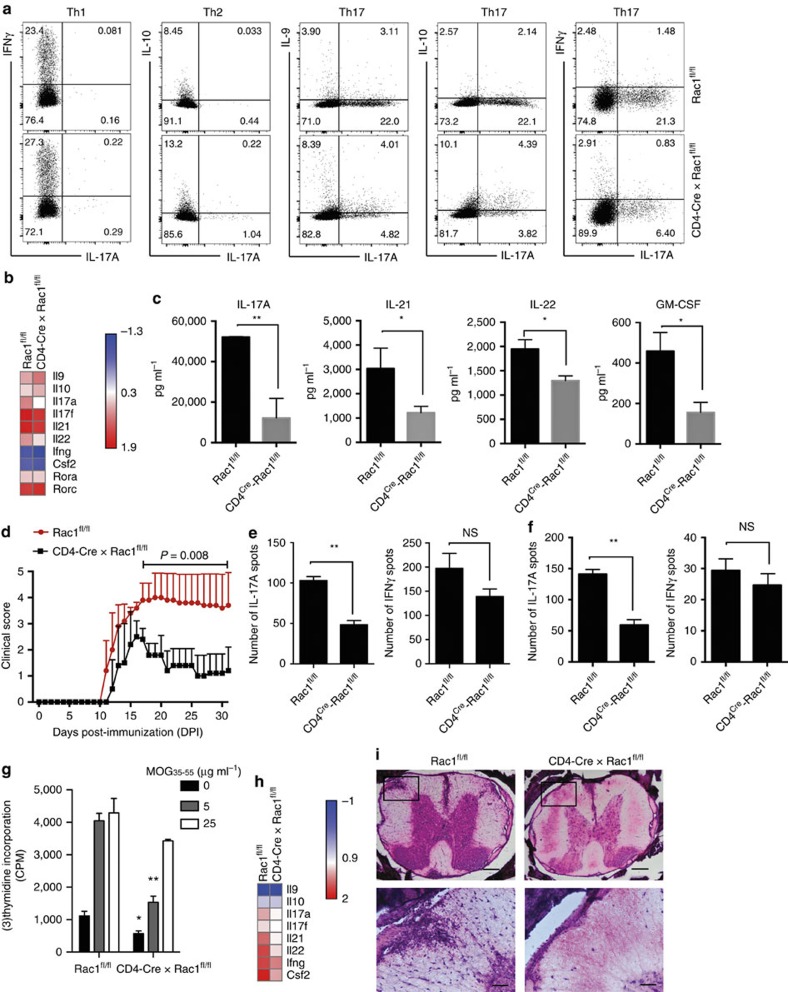
Rac1 genetic deficiency reduces IL-17A expression and improves EAE symptoms. (**a**) Naive CD4^+^ T cells were isolated from mice with conditional deletion of Rac1 in CD4^+^ T cells (*CD4-Cre x Rac1*^fl/fl^) or control floxed mice (*Rac1*^fl/fl^) and T cells were stimulated under Th1, Th2 and Th17 conditions and cytokine profile was analyzed by intracellular staining. (**b**) Heat map of relative mRNA expression of Th17-associated molecules by Taqman PCR from *CD4-Cre x Rac1*^fl/fl^ and control *Rac1*^fl/fl^ mice. (**c**) Cytokine expression by Luminex. Naive CD4^+^ T cells were isolated from *CD4-Cre x Rac1*^fl/fl^ or control *Rac1*^fl/fl^ mice and T cells were stimulated *in vitro* under pathogenic Th17 cell condition with IL-6 and TGF-β1 for 4 days, rested for 2 days, and followed by restimulation with the addition of IL-23 for another 2 days. (**d**) Mean EAE clinical score (±s.e.m.) measured in CD4-*Cre x Rac1*^fl/fl^ and littermate control *Rac1*^fl/fl^ mice (*n*=10 mice per group) that were immunized with MOG_35–55_/CFA. The mean maximal score of *CD4-Cre x Rac1*^fl/fl^ versus control *Rac1*^fl/fl^ mice was compared by two-tailed Mann–Whitney *U* test, *P*=0.008. (**e**,**f**) ELISPOT analysis of the frequency of IL-17A and IFNγ-producing cells out of 400,000 (**e**) spleen and (**f**) LN cells isolated from *CD4-Cre x Rac1*^fl/fl^ and *Rac1*^fl/fl^ immunized mice that were re-challenged with MOG_35–55_ peptide (10 μg ml^−1^) for 36 h and positive cells were quantified (*n*=3–5 mice per group). (**g**) MOG_35–55_-specific proliferation of splenocytes that were isolated from *CD4-Cre x Rac1*^fl/fl^ and *Rac1*^fl/fl^ mice and re-challenged with MOG_35–55_ peptide (0, 5 and 25 μg ml^−1^) 10 days after mouse immunization. (**h**) Heat map showing relative cytokine expression of CNS-infiltrated CD4^+^ T cells. Infiltrated cells were prepared from the spinal cords of recipient mice (3 mice per group) followed by Taqman PCR. (**i**) Histopathology of H&E-stained spinal cord sections of mice described in **d** on DPI 14. Boxed area in *top row* is enlarged below. Original magnification × 10 (*top row*) and × 40 (*bottom row*) with indicated calibration bars corresponding to 200 μm (*top row*) and 50 μm (*bottom row*). Results are representative of two independent experiments with two mice per group. Data are shown as mean±s.e.m.*P<0.05; **P<0.01 by Student *t*-test. *NS*, non-significant.

GEF's such as Tiam1 are required for activation of the GTPase Rac1 that has been implicated in a myriad of cellular processes. We investigated the role of Rac1 in the generation of Th17 cells *in vitro* and *in vivo*. Floxed Rac1 (*Rac1*^fl/fl^) mice were crossed with CD4-Cre syngeneic mice to generate animals with conditional deletion of Rac1 in CD4^+^ T cells (*CD4-Cre x Rac1*^fl/fl^). Naive CD4^+^ T cells from *CD4-Cre x Rac1*^fl/fl^ as well as *Rac1*^fl/fl^ control mice were differentiated for 4 days under Th1, Th2 and Th17 cell polarization conditions. Interestingly, CD4^+^ cells from *CD4-Cre x Rac1*^fl/fl^ mice had a specific and significant decrease in IL-17A production accompanied by an increase in IL-9 and IL-10 frequency under Th17 conditions induced by IL-6 plus TGF-β1, with no significant change in cytokines under Th1 and Th2 polarizing conditions (Fig. 3a). Further analysis of cytokine expression of differentiated Th17 cells by Taqman PCR confirmed a marked decrease in *Il17a* expression that was associated with an increase in *Il9* and to a lesser extent *Il10* expression. Rac1 deficiency in T cells does not modulate *Rora*/*Rorc* or other Th17-associated molecules (Fig. 3b). To test the effects of Rac1 on the differentiation of Th17 cells in the presence of IL-23, naive CD4^+^ T cells from Rac1-deficient mice and control littermates were differentiated for 4 days under Th17 conditions of IL-6 plus TGF-β1, rested for 2 days, and then cultured in the presence of recombinant IL-23 for 2 more days. Cytokine analysis of the culture supernatants showed that Rac1 deletion reduced significantly IL-17A in addition to a reduction in IL-21, IL-22 and GM-CSF (Fig. 3c). In addition, we showed that Th17 cells with Rac1 deficiency differentiated using IL-1β, IL-6, and IL-23 exhibited a decrease in IL-17A, IL-17F, IL-22 and IL-23 receptor expression (Supplementary Fig. 3).

Next, we examined the function of Rac1 in EAE mice. We found that clinical scores in *CD4-Cre x Rac1*^fl/fl^ mice were significantly reduced compared with *Rac1*^fl/fl^ control littermates. The mean maximal score of *CD4-Cre x Rac1*^fl/fl^ mice was 2.5±0.3 versus 4.1±0.4 in control *Rac1*^fl/fl^, *P*=0.008 by Mann–Whitney *U* test (Fig. 3d). Analysis of cytokine production of MOG_35–55_-reactive CD4^+^ T cells in spleen and LN tissues by ELISPOT analysis showed a significant decrease in IL-17A-reactive cells in *CD4-Cre x Rac1*^fl/fl^ mice compared with control mice while IFNγ production was not significantly changed (Fig. 3e for spleen and Fig. 3f for LN). A panel of cytokines was measured by Luminex in spleen and LN cells treated with MOG_35–55_ peptide, which further demonstrates the involvement of Rac1 in IL-17A production in addition to other Th17-associated cytokines such as IL-21, IL-22 and GM-CSF (Supplementary Fig. 4). Although not significant, a slight increase in IL-9 expression was observed in Rac1 deficient mice, suggesting that IL-9 production is not a major player in the disease course of *CD4-Cre x Rac1*^fl/fl^ mice (Supplementary Fig. 4). Finally, we found that Rac1 deletion reduced the ability of MOG_35–55_ stimulation to induce T-cell proliferation (Fig. 3g).

To analyze the cytokine profile of CD4^+^ T cells in the central nervous system (CNS) of EAE mice, spinal cord tissues were collected 12 days after immunization, and infiltrated cells were isolated and processed for cytokine expression by Taqman PCR. Our findings confirm that Rac1 deletion causes a decrease in the expression of *Il17a*, *Il21* and *Il22*. Interestingly, a decrease in *Csf2* (gene encoding for GM-CSF) and to a lesser extent *Ifng* was detected in the spines of *CD4-Cre x Rac1*^fl/fl^ mice (Fig. 3h). In addition, histological examination revealed marked decrease in inflammatory infiltrates in spinal cord sections of *CD4-Cre x Rac1*^fl/fl^ mice compared with control *Rac1*^fl/fl^ mice (Fig. 3i).

### Tiam1/Rac1 inhibition reduces IL-17A and EAE symptoms

**Figure 4:**
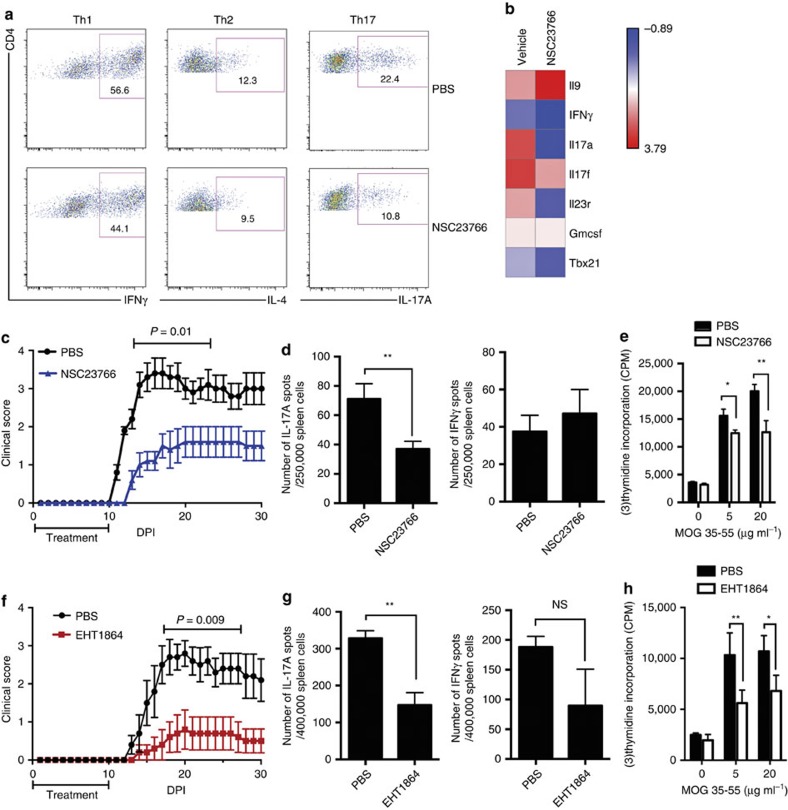
Pharmacological inhibition of Tiam1/Rac1 reduces murine IL-17A expression and ameliorates EAE symptoms. (**a**) Flow cytometry staining of CD4^+^ T cells treated with NSC23766. Naive CD4^+^ T cells were isolated from WT mice and were polarized under Th1, Th2 and Th17 cell conditions in the presence of NSC23766 (94 μM) or control solvent. Cytokine expression was measured on day 4 after differentiation. Numbers in outlined areas indicate percentage of positive cells. (**b**) Heat map of mRNA expression of Th17-associated cytokines in Th17 cells treated with NSC23766. Naive CD4^+^ T cells were differentiated *in vitro* with IL-6 and TGF-β1 in the presence or absence of NSC23766 for 4 days and cytokine profile was assessed by Taqman PCR. (**c**,**f**) Mean EAE clinical score (±s.e.m.) measured in WT mice that received NSC23766 (**c**), EHT1864 (**f**) or solvent (*n*=15–20 mice per group). Mice were immunized with MOG_35–55_/CFA and received 8 daily injections of (**c**) NSC23766 (5 mg kg^−1^) or (**f**) EHT1864 (40 mg kg^−1^) i.p. starting on the day of immunization. Control mice received PBS treatment according to the same regimen. (**d**,**g**) ELISPOT assay of the frequency of MOG_35–55_-reactive IL-17A and IFNγ-producing cells in immunized mice. Spleens were isolated from NSC23766 (**d**) or EHT1864 (**g**) and control mice on day 10 after immunization and were stimulated with MOG_35–55_ peptide (10 μg ml^−1^) for 36 h and positive cells were quantified (*n*=3–5 mice per group). (**e**,**h**) MOG_35–55_-specific proliferation of splenocytes isolated from NSC23766 (**e**) or EHT1864 (**h**) and control mice on day 10 after immunization. Cultures were re-challenged with MOG_35–55_ peptide (0, 5 and 25 μg ml^−1^) and proliferation was measured by [^3^H]-thymidine incorporation reported as CPM (+s.d.) from triplicate wells (*n*=3–5 mice per group). Data are representative of three experiments with similar results and are shown as mean±s.e.m. *P<0.05; **P<0.01 by Student *t*-test. NS, non-significant.

Our findings suggest that targeting the *Tiam1*/*Rac1* complex is a viable approach for regulating Th17 cell-mediated autoimmune encephalomyelitis *in vivo*. We tested Tiam1/Rac1 inhibition in Th17 cells polarized with IL-6 plus TGF-β1 *in vitro* using NSC23766, a small molecule that effectively inhibits the Rac1-specific GEF, Tiam1, from binding and activating Rac1 (ref. 34). Our results showed that NSC23766-treated Th17 cells had a marked reduction of IL-17 producing T cells (Fig. 4a). Analysis of Th17 cells by Taqman showed a decrease in the mRNA expression of IL-17A, IL-17F and IL-23R, whereas IL-9 expression was elevated. Treatment with NSC23766 did not affect IFNγ, Tbx21 and GM-CSF expression that were weakly expressed in Th17 cells differentiated with IL-6 plus TGF-β1 (Fig. 4b). There was no effect of NSC23766 on cytokine expression by Th1, or Th2 cells (Fig. 4a). We did not observe any modulation of cell activation or survival of CD4^+^ T cells treated *in vitro* with NSC23766 (Supplementary Fig. 5A,B).

*In vivo*, administration of eight consecutive injections of NSC23766 (5 mg kg^−1^, i.p. daily) daily i.p. during the priming phase of EAE (days 0–7 post-immunization) alleviated clinical disease (mean maximal score of NSC23766-treated mice 1.9±0.4 versus 3.7±0.3 in control phosphate-buffered saline (PBS) recipients, *P*=0.01 by two-tailed Mann–Whitney *U* test) (Fig. 4c). The protective effects of NSC23766 were accompanied by a significant decrease in MOG_35–55_-reactive IL-17A-producing CD4^+^ T cells measured in the splenocytes by ELISPOT with no significance alterations of IFNγ-producing T cells (Fig. 4d). To measure the influence of NSC23766 on the magnitude of MOG_35–55_-specific T-cell response, we measured splenocyte proliferation of control and treated mice. Splenocytes were re-challenged *in vitro* with increasing doses of MOG_35–55_ peptide (0–20 μg ml^−1^) and cell proliferation was measured 2 days later. We found that NSC2366-treated mice exhibit decreased cell proliferation (Fig. 4e). Given that Tiam1/Rac1 signalling is implicated in cell migration, we observed a notable reduction in CD4^+^ T-cell infiltration in the spinal cord tissues in mice that received NSC23766 treatment. This suggests that in addition to the reduction in IL-17^+^ CD4^+^ T cells by NSC23766 treatment, decreased T-cell infiltration may contribute to the protective effects of NSC23766 in EAE (Supplementary Fig. 6).

To confirm the protective effects of Rac1 pharmacological inhibition in EAE, we utilized EHT1864 another small-molecular weight compound that directly inhibits active Rac1 (ref. 35). Mice received eight consecutive injections of EHT1864 (40 mg kg^−1^) every day according to the regimen described above and were monitored for clinical symptoms for up to 30 days after immunization. As expected, inhibition of Rac1 activity ameliorated disease severity confirming the therapeutic effects of targeting Rac1 in EAE (mean maximal score of EHT1864-treated mice 1.7±0.4 versus 3.4±0.3 in control PBS recipients, *P*=0.009 by two-tailed Mann–Whitney *U* test) (Fig. 4f). In agreement with the data reported in the Rac1-deficient mice, we found that pharmacological inhibition of Rac1 activation decreased significantly MOG_35–55_-reactive IL-17A-producing CD4^+^ T cells as measured in the splenocytes by ELISPOT with no significant alterations of IFNγ-producing T cells (Fig. 4g). To measure the influence of EHT1864 on the magnitude of MOG_35–55_-specific T-cell response, we measured splenocyte proliferation of control and treated mice. We found that splenocytes from EHT1864-treated mice exhibited decreased cell proliferation (Fig. 4h).

### Tiam1/Rac1 and RORγt cooperatively bind to Il17 promoter

**Figure 5:**
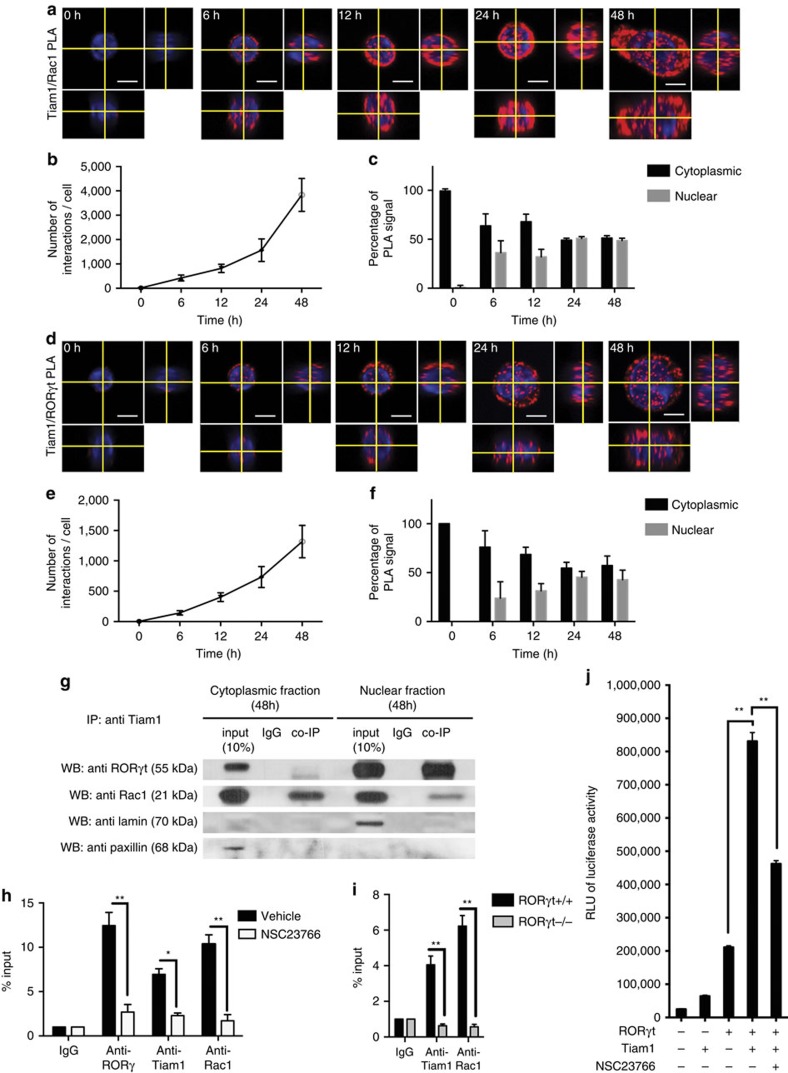
Physical interaction between Tiam1, Rac1 and RORγt and nuclear translocation in murine Th17 cells. (**a**) Time course of Tiam1/Rac1 interaction as detected by PLA during Th17 cell differentiation. Each red dot represents a single Tiam1/Rac1 molecular interaction. (**b**) Quantification of the number of Tiam1/Rac1 interactions per cell in the course of Th17 cell differentiation (*n*=10 cells for each time point). (**c**) Assessment of the subcellular distribution of Tiam1/Rac1 PLA signals in differentiating Th17 cells. (**d**) Interaction between Tiam1 and RORγt as detected by PLA at different stages of Th17 cell differentiation. The calibration bars in (**a**,**d**) represent 5 μm. (**e**) Quantification of the number of Tiam1/RORγt interactions per cell during the course of Th17 cell differentiation. (**f**) Assessment of the subcellular distribution of Tiam1/RORγt PLA signals in Th17 cells. (**g**) Co-IP analysis of the interaction of Tiam1 with Rac1 and RORγt in fractionated Th17 cells (48 h). Paxillin (cytoplasmic protein) and lamin (nuclear protein) served as controls for the fractionation. (**h**) ChIP-PCR analysis of Tiam1 and Rac1 binding to the *Il17* promoter. Naive CD4^+^ T cells were stimulated under Th17 cell conditions±NSC23766 for 2 days. Total input DNA before IP was used for normalization of data. The graphs represent quantitative PCR analysis of the ratio of enriched *Il17* promoter with RORγt binding sites to the input DNA. (**i**) ChIP-PCR analysis of Tiam1 and Rac1 binding to the *Il17* promoter in naive CD4^+^ cells isolated from RORγt*−*/− and control RORγt+/+ mice differentiated for 2 days under Th17 conditions. Data are presented as average±s.e.m. of per cent input with subtraction of control IgG. (**j**) *Il17* luciferase reporter assay. HEK 293T cells were transfected with pGL3-CNS2-Il17 vectors in the presence of the indicated constructs, cells were cultured for 24 h±NSC23766, protein extracts were prepared, and luminescence was measured. Data represent mean±s.e.m. of a representative experiment each performed in triplicate. *P<0.05; **P<0.01 by Student *t*-test.

Differentiation of Th17 cells is controlled by the master transcription factor, RORγt, which dictates a specific and heritable gene expression profile^13^. Given that Rac1 has been shown to modulate gene expression in various cell types^23^, we hypothesized that the Tiam1/Rac1 complex may regulate Th17 cell differentiation by direct interaction with RORγt. We performed temporal and spatial protein–protein binding studies in Th17 cells on per cell basis using proximity ligation assay (PLA), a very sensitive technique to measure protein–protein interaction as detailed in ‘Methods' section. First, we found that Tiam1 and Rac1 interact physically in Th17 cells starting at 6 h and this interaction is further enhanced at 24 and 48 h after differentiation, in both the cytoplasmic and nuclear compartments (Fig. 5a–c). Interestingly, we found that Tiam1 interacting with RORγt in Th17 cells was detected mostly in the cytoplasm 6 h after differentiation followed by accumulation of the Tiam1/RORγt complex in the both the nuclei and cytoplasm of Th17 cells at 24 and 48 h (Fig. 5d–f)

In a second set of experiments, co-immunoprecipitation (co-IP) studies were performed using cytoplasmic and nuclear extracts from Th17 cells polarized for 48 h to confirm the observed interaction between endogenous Tiam1/Rac1 and RORγt. Precipitation with anti-Tiam1 antibody yielded bands corresponding to both Rac1 and RORγt, whereas precipitation with the nonspecific IgG antibody serving as the negative control yielded neither band (Fig. 5g; Supplementary Fig. 7). Specifically, we observed a strong interaction between Tiam1 and Rac1 in both extracts whereas Tiam1/RORγt interaction was mainly detected in the nuclear extracts. Taken together, our findings demonstrate the existence of a Tiam1*–*Rac1–RORγt complex in Th17 cells.

Next, we assessed whether the Tiam1/Rac1 complex is recruited to the *Il17* promoter in Th17 cells. Naive CD4^+^ T cells were differentiated for 48 h and chromatin immunoprecipitation followed by PCR (ChIP-PCR) analysis was used to measure a possible recruitment of Tiam1 and/or Rac1 to the conserved non-coding sequence (CNS2) of *Il17* promoter, a *cis* element that physically interacts with both *Il17a* and *Il17f* gene promoters and is required for RORγt-driven IL-17 production in Th17 cells^36^. ChIP assay using PCR primers encompassing the *CNS2* region demonstrated the suspected dual involvement of Tiam1 and Rac1 in RORγt-mediated binding specifically to the CNS2 of the *Il17* promoter (Fig. 5h). First, we found that both Tiam1 and Rac1 are present at the CNS2 48 h after Th17 cell differentiation. Note the high degree of Tiam1/Rac1 nuclear translocation at the same time point (Fig. 5a,g). Second, pharmacological inhibition of Rac1 using NSC23766 not only abolished Tiam1 and Rac1 binding to the CNS2 but also it has hindered RORγt recruitment to the *Il17* promoter (Fig. 5h). Third, using conditional RORc null mice (*CD4-Cre x RORc*^fl/fl^), we found that the recruitment of Tiam1/Rac1 complex to CNS2 is completely abolished in the absence of RORγt in Th17 cells (Fig. 5i). Altogether, these findings suggest that Tiam1/Rac1 nuclear translocation and interaction with the CNS2 region in Th17 cells is RORγt-dependent. To analyze the functional relevance of Tiam1/Rac1 physical interaction with RORγt in Th17 cells, we investigated the ability of Tiam1-mediated Rac1 activation to induce the *Il17* promoter in reporter assays using 293T cells. We used the reporter construct pGL3-CNS2, containing the firefly luciferase gene under the control of the *Il17* promoter described previously^36^. Co-transfection of the pGL3-CNS2 luciferase reporter construct with a plasmid encoding the N-terminal truncated mutant of Tiam1 (C1199-Tiam1), a Tiam1 variant that has been shown to be more stable and more active than the full length^37^, resulted in a significant amplification of RORγt-induced *Il17* promoter activation (*P*<0.01 by unpaired Student's *t*-test) (Fig. 5j). This was significantly but not totally inhibited when transfected cells were treated with the Rac1 inhibitor NSC23766 (*P*<0.01 by unpaired Student's *t*-test) (Fig. 5j). The fact that NSC23766 only partially inhibits Tiam1-mediated *Il17* promoter activation suggests the other GEFs may be involved in the induction of Rac1 activation in this system. The specific involvement of Tiam1 in the enhancement of RORγt-induced *Il17* promoter activation is further verified by the lack of Tiam1-mediated regulation of the function of T-bet, the master transcription factor of Th1 cells. Indeed, we found that Tiam1 does not regulate *Ifng* promoter activation when overexpressed alone or in the presence of T-bet in a luciferase reporter assay (Supplementary Fig. 8). This is in agreement with the findings that inhibition of Tiam1 and Rac1 in Th1 cells exhibited only modest effects on IFNγ production (Figs 2a and 3a).

### Human Tiam1/Rac1 pharmacological inhibition decreases IL-17A

**Figure 6:**
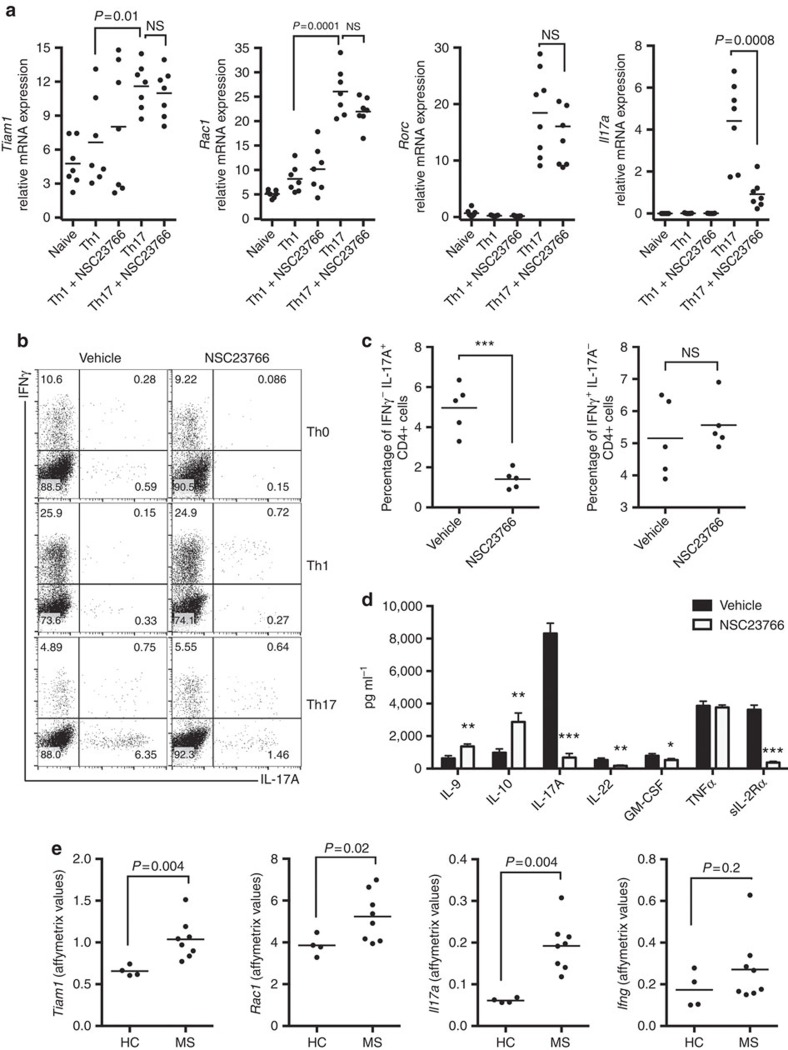
Pharmacological inhibition of Tiam1/Rac1 signaling regulates human Th17 Cells. (**a**) Tiam1 and Rac1 expression is induced in human Th17 cells. Naïve CD4^+^ T cells were isolated from fresh PBMC obtained from healthy subjects (*n*=7) by MACS negative selection and cells were cultured under Th1 and Th17 cell polarizing conditions in the presence or absence of NSC23766 (94 μM) for 7 days. Tiam1 and Rac1 expression was analyzed by Taqman PCR. RORγt and IL-17A mRNA levels are shown as controls for polarization. (**b**) Cytokine expression of NSC23766-treated Th17 cells by flow cytometry. Naive CD4^+^ T cells were polarized under Th0, Th1, and Th17 conditions in the presence or absence of NSC23766 for 7 days followed by activation for 4 h with PMA and ionomycin. Cells were fixed, permeabilized then incubated with fluorochrome-conjugated anti-IL-17A (*x* axis) and anti-IFNγ (*y* axis) antibodies. Numbers in outlined areas indicate per cent of positive cells. (**c**) Mean of the frequency of IL-17A^+^IFNγ^−^ and IL-17A^−^IFNγ^+^ in Th17 cells starting from naïve CD4^+^ T cells isolated from healthy subjects (*n*=5) and differentiated in the presence or absence of NSC23766 is shown. (**d**) Cytokine profile of NSC23766-treated Th17 cells by Luminex. Supernatants were collected from Th17 cell cultures at the end of the differentiation and were analyzed by bead-based Luminex assay according to the manufacturer's instructions. Data represent mean±s.e.m. of a representative experiment each performed in triplicate. (**e**) Tiam1 and Rac1 expression in MS patients. Gene expression profiling of CD4^+^ T cells from MS patients (*n*=8) and healthy controls (HC) (*n*=4) analyzed by Affymetrix gene array and deposited on GEO (GSE32988). *P<0.05; **P<0.01; ***P<0.001 by Student *t*-test. Each dot represents an individual. *NS*, non-significant.

Our findings that Rac1 inhibition suppressed murine Th17 cell differentiation and EAE development led us to question whether Rac1 signalling is also involved in human T-cell development. Therefore, we analyzed Tiam1 and Rac1 expression in human Th17 cells. Compared with Th1 cells, we found that the expression of both Tiam1 and Rac1 is significantly higher in Th17 cells differentiated *in vitro* according to a standard protocol for 7 days. In addition, pharmacological inhibition of Tiam1/Rac1 activity using NSC23766 reduced IL-17A mRNA expression in Th17 cells without regulating *Rorc* gene level suggesting that NSC23766 effects are independent of RORγt expression (Fig. 6a). To further evaluate the role of Tiam1/Rac1 in T cells, CD4^+^ T cells were differentiated under Th1 and Th17 cell conditions in the presence or absence of NSC23766. Analysis of IL-17A expression by flow cytometry demonstrated that NSC23766 specifically regulates IL-17A expression in Th17 cells while sparing IFNγ in both Th1 and Th17 cells (Fig. 6b,c; Supplementary Fig. 9A). In agreement with the data generated in mouse Th17 cells, NSC23766 downregulated the production of IL-22 and GM-CSF in addition to inducing a robust decrease in IL-17A. In contrast, addition of NSC23766 upregulated IL-9 and IL-10 as measured by bead-based Luminex assay in Th17 cell cultures (Fig. 6d). Notably, we detected a significant decrease in soluble IL-2Rα, a pro-inflammatory cytokine that has been shown to enhance the development of Th17 cells and to exacerbate autoimmunity in EAE mice^38^ (Fig. 6d). To determine whether Tiam1 and Rac1 expression is altered in the inflammatory conditions of MS patients, we extracted gene microarray data of CD4^+^ T cells from eight MS patients and four healthy controls deposited on GEO (accession code GSE32988)^39^. Strikingly, there was a significant increase in Tiam1 and Rac1 gene expression in CD4^+^ T cells from MS patients compared with healthy controls (Fig. 6e). Of interest, we noticed a dominant Th17 cytokine profile in this group of MS patients as revealed by increased expression of *Il17a*, *Il17f, Il21*, *IL23r* and *Rorc* (Fig. 6e; Supplementary Fig. 9B). These findings further highlight a possible involvement of Tiam1/Rac1 signalling in Th17 cell development in MS patients.

## Discussion

The master transcription factor of Th17 cells, RORγt, is a well characterized drug target that holds promise for autoimmune disease therapy. Although several groups have reported on small molecule inhibitors of RORγt that are capable of binding to its LBD and suppressing *Il17* transcription *in vitro* and *in vivo*^18^,^21^, the regulation of this orphan nuclear receptor by endogenous proteins has yet to be determined. Our present study identifies a novel signalling pathway where the Tiam1/Rac1 complex regulates RORγt function in Th17 cells.

Most of the cellular functions attributed to the Tiam1/Rac1 complex depend on its spatio-temporal-controlled activation. Whereas it is generally accepted that Tiam1/Rac1 signals at the plasma membrane or on intracellular vesicles, there is increasing evidence for GTPase signalling in the nucleus^40^,^41^. This is further supported by the discovery that some GTPases such as Rac1 possess a nuclear localization signal raising the possibility that Rac1 may play an important role not only in the cytosol but in the nucleus as well^31^. Our confocal and ChIP analyses provide novel evidence that both Tiam1 and Rac1 are induced in the cytoplasm and translocate to the nucleus in Th17 cells where they are recruited to the *Il17* promoter in a RORγt-dependent manner as evidenced by the use of RORc-deficient Th17 cells. Protein translocation across the nuclear membrane occurs through nuclear pore complexes that assume the role of gatekeepers. Small molecules (<40 kD) such as Rac1 are able to pass through the nuclear pore by diffusion, while active nuclear import of protein complexes such as Tiam1/Rac1 is mediated by karyopherins^42^. Whether RORγt is playing a direct role in the nuclear translocation of Rac1/Tiam1 complex remains to be determined.

Given the large number of protein interaction domains found in its structure, Tiam1 has been described to associate with a myriad of membrane and intracellular proteins such as CD44 (ref. 43), spinophilin^44^, and JIP/IB2 complex^45^ thus regulating downstream gene transcription. These reported findings are in agreement with our present study of Th17 cells where Tiam1 co-localizes with RORγt in the nucleus of Th17 cells as we demonstrated by confocal images and further confirmed by ChIP data at the *Il17* promoter level.

Pathogenic subpopulations of Th17 cells are characterized by co-expression of RORγt and T-bet, co-produce IL-17 and IFN-γ, and drive autoimmunity in mice^46^. They are distinct from ‘classical' Th17 cells that are not pathogenic, and produce mainly IL-17, IL-9 and IL-10 (ref. 15). We find that inhibition of Tiam1/Rac1 activation regulates the cytokine profile of Th17 cells as evidenced by a down-regulation of IL-17A and an up-regulation in IL-9 and IL-10 expression at the gene and protein levels. Previously, we have reported that Th17 cells lacking RORγt expression exhibit a cytokine signature similar to that found in non-pathogenic Th17 cells, namely a decrease in IL-17A and IL-17F and an increase in IL-9 and IL-10 expression^20^. Our present *in vivo* findings showing amelioration of EAE clinical disease in response to genetic deletion or pharmacological inhibition of Tiam1/Rac1 activity suggest a potential role of Tiam1/Rac1 not only in the generation of IL-17A-producing CD4^+^ T cells, (IL-17 being a key cytokine required for EAE induction^47^), but also in potentiating their encephalitogenicity. Given that inhibition of the Tiam1/Rac1 axis confers a similar phenotype to that reported in RORγt-deficient Th17 cells, further supports that RORγt downstream signalling is a critical target of the Tiam1/Rac1 complex.

Having been mostly investigated in the context of neoplasms, targeting the Tiam1/Rac1 complex either genetically or using pharmacological inhibitors has shown the ability to deter proliferative ability of several tumours^30^,^48^ in addition to primary cells^49^,^50^. The effects of Rac1 on Th17 cell development are postulated to go beyond the transcriptional effects on IL-17A to encompass the ability of these cells to migrate and form efficient immune synapses with APCs. The pronounced anti-proliferative effects mediated by genetic deficiency or pharmacological inhibition of Tiam1/Rac1 complex might be rooted in the hindered ability of T cells to establish optimal contact with APCs, which is needed to perpetuate T-cell receptor activation and cellular proliferation. Rho-GTPases including Rac1 have been shown to play a crucial role in cytoskeletal remodelling that mediates several cellular processes such as cellular migration, polarization and efficient immunological synapse formation^51^. As such, active Rac1 orchestrates actin polymerization needed for the formation of lamellopodia, fillopodia and microvilli in several cell types. Equally important is Rac1's ability to decrease cellular stiffness via the resorption of these cellular processes to ensure proper cell–cell contact. On antigen recognition and chemokine stimulation of lymphocytes, Rac1 promotes dephosphorylation of the ERM family (ezrin/radixin/moesin), which is known to cross link actin filaments with the plasma membrane^52^. These cellular events diminish T-cell membrane rigidity allowing the dynamic resorption of cellular processes and optimal subsequent cellular contact with APCs. Therefore, investigating the effect of genetic deficiency or pharmacological inhibition of the Tiam1/Rac1 complex on antigen recall, it is expected to see a weakened antigenic recognition ability as evidenced by the dampened proliferation of cultured splenocytes from MOG_35–55_ immunized mice that were treated with NSC23766 and EHT1864 *in vivo*.

In conclusion, we have demonstrated that Tiam1/Rac1 signalling is required for the encephalitogenicity of CD4^+^ T cells, very likely through its regulation of RORγt-mediated *Il17* transcription. Our work herein demonstrates that the Tiam1/Rac1 complex controls this cytokine transcriptionally, but more investigation will be required to further characterize the molecular details of this regulation. Moreover, the question of whether Tiam1/Rac1 signalling in T cells influences the pathogenesis of other autoimmune or infectious diseases is intriguing.

## Methods

### Animals and EAE induction with MOG_35–55_

Six- to eight-week-old female WT C57BL/6 mice were purchased from The Jackson Laboratory. *Tiam1−*/− (Knockout) mice^27^ were provided by John G. Collard (Divisions of Cell Biology, The Netherlands Cancer Institute, Amsterdam, The Netherlands). The Tiam1 knockout mice were backcrossed repeatedly (8 × ) to FVB mice in the lab of origin (Collard lab). Subsequently, the mice has been backcrossed with C57BL/6 for 8 generations in our lab. C57BL/6, *Rac1*^fl/fl^ (ref. 53) (on a mixed 129S4/SvJae, BALB/c, C57BL/6 background), and *Rorc*^fl/fl^ mice^54^ (on a mixed C57BL/6J:C67/BL6N genetic background) were purchased from The Jackson Laboratory and were bred with *CD4-Cre* transgenic mice (Taconic) for obtaining T-cell-specific Rac1-Rac1-null and RORγ-RORγt-null mice, respectively. STAT3 floxed mice (on C57BL/6 background) were previously described^55^ and were crossed with *CD4-Cre* transgenic mice. EAE was induced by subcutaneously immunizing mice in the flanks with myelin oligodendrocyte glycoprotein peptide (MOG)_35–55_ (New England Peptide). The 100 μg of administered (MOG)_35–55_ was comprised of 50 μl PBS and 50 μl CFA containing 250 ng *Myobacterium tuberculosis* (Fisher Scientific). Intraperitoneal injection of 200 ng pertussis toxin (List Biological Laboratories) followed on the day of immunization as well as 2 days later.

### Adoptive transfer and treatment with Tiam1/Rac1 inhibitors

For the adoptive transfer of *Tiam1−*/− T cells, *Tiam1−*/− and *Tiam1+*/+ mice were immunized with MOG_35–55_/CFA for 8 days, draining LN cells were isolated and re-activated with MOG_35–55_ peptides (20 μg) in the presence of mouse recombinant IL-23 (20 ng ml^−1^) for 2 days, CD4^+^ T cells were isolated by MACS magnetic beads and 0.75 million cells were injected i.p. in Rag1 deficient mice. Recipient mice received PT injections (69 ng) on days 1 and 3 after cell transfer. For Rac1 pharmacological inhibition, WT mice received 5 mg kg^−1^ of NSC23766 (Calbiochem), 40 mg kg^−1^ of EHT 1864 (TOCRIS) or PBS on the day of the immunization and then daily for 7 days. Animals were kept for observation and scoring for 30 days. EAE clinical disease was scored accordingly: score 1, limp tail or isolated gait weakness without limp tail; score 2, gait weakness or partial hind and/or front limb paralysis; score 3, total hind limb paralysis; score 4, total hind limb and partial front limb paralysis; score 5, moribund animal or death. Mice were housed in the New Research Building Animal Facility at Harvard Medical School under specific pathogen-free conditions. All experiments involving animals were done with the approval of the Harvard Medical Area Standing Committee on Animals and in compliance with their protocols.

### Antibodies and reagents

All FACS antibodies (Abs) and blocking Abs were purchased from BD Biosciences or eBioscience. Mouse anti β-actin monoclonal antibody (mAb), rabbit anti-Tiam1 antibody, and mouse anti-Rac1 antibody used for western blot were purchased from Sigma-Aldrich, Millipore and BD Biosciences, respectively. Mouse monoclonal Tiam1 antibody from Santa Cruz Biotechnology was used for immunoprecipitation. Other antibodies used for co-IP include mouse anti RORγ (BD Biosciences), mouse anti-Rac1 (Abcam), mouse anti lamin A/C (Cell Signaling) and goat anti paxillin (Santa Cruz Biotechnology). For the proximal ligation assay, rabbit anti-Tiam1 (Santa Cruz Biotechnology), mouse anti-Rac1 (Abcam), mouse anti RORγ (BD Biosciences) were used. All recombinant cytokines were purchased from R&D Systems.

### Mouse and human T-cell differentiation

Naive CD4^+^ T cells were purified from naive C57BL/6 mice using magnetic-activated cell sorting (MACS) (Miltenyi). Cells (250–500 × 10^3^) were stimulated using plate-bound anti-CD3 (4 μg ml^−1^) as well as soluble anti-CD28 (2 μg ml^−1^) in 48-well plates for 4 days in a serum-free medium (X-VIVO-20; Lonza) supplemented with 50 μM 2-mercaptoethanol, 1 mM sodium pyruvate, L-glutamine, nonessential amino acids, and 100 U ml^−1^ of penicillin and streptomycin in the presence of recombinant cytokines. Naive CD4^+^ T cells were polarized in the presence of recombinant mouse IL-12 (10 ng ml^−1^) and anti-IL-4 neutralizing antibody (10 μg ml^−1^) for Th1, recombinant mouse IL-4 (10 ng ml^−1^) plus anti-IFNγ antibody (10 μg ml^−1^) for Th2, recombinant mouse IL-6 (30 ng ml^−1^) and recombinant human TGF-β1 (3 ng ml^−1^) plus anti-IFNγ (10 μg ml^−1^) for Th17. For pathogenic Th17 cells, recombinant IL-1β and IL-23 were used at 20 ng ml^−1^ when cells were polarized using IL-6, IL-1β and IL-23 during 4 days of culture. In addition, pathogenic Th17 cells were alternatively polarized with IL-23 at 10 ng ml^−1^ for 48 h in the presence of IL-6 and TGF- β1 used to restimulate differentiated Th17 cells that were rested prior for 2 days in the presence of IL-2 (2 ng ml^−1^). No recombinant cytokines or blocking Abs were added for Th0 condition. NSC23766 (94 μM) was added when indicated.

For human T-cell studies, blood was drawn from healthy control volunteers in compliance with the Institutional Review Board protocols. Ficoll-Paque PLUS (GE Healthcare) was used to separate PBMCs by gradient centrifugation. Naive CD4^+^ T cells were subsequently isolated from fresh PBMCs by negative selection with beads using the CD4^+^ T-cell isolation kit II (Miltenyi). Cells were cultured at 10^4^ cells per well in serum-free X-Vivo 15 media (Lonza), and stimulated for 7 days with plate-bound anti-CD3 (UCHT1, 5 μg ml^−1^) and soluble anti-CD28 (28.2, 1 μg ml^−1^) Abs in the presence of anti-IFN-γ plus anti-IL-4 for Th0, IL-12 (10 ng ml^−1^) plus anti-IL-4 for T helper 1, or TGF-β1 (5 ng ml^−1^), IL-6, IL-21, IL-23 (all at 25 ng ml^−1^) and IL-1β (12.5 ng ml^−1^) for T helper 17 cells. Neutralizing Abs were used at 10 μg ml^−1^. All recombinant proteins were purchased from R&D Systems. Neutralizing antibodies were purchased from BD Biosciences.

### Flow cytometry

For intracellular cytokine staining, *in vitro* polarized cells or cells isolated from mice LNs, spleen or spinal cords were washed then stimulated in culture medium with phorbol 12-myristate 13-acetate (PMA) (50 ng ml^−1^; Sigma-Aldrich) and ionomycin (300 ng ml^−1^; Sigma-Aldrich) for 4 h in the presence of GolgiStop (BD Biosciences) at 37 °C, in a humidified 5% CO_2_ chamber. The cells were then washed and stained for 20 min with 7AAD for dead cells exclusion and fluorochrome-labelled mAbs against surface cell markers. The cells were fixed and permeabilized using Cytofix/Cytoperm (BD Biosciences) and Perm/Wash buffer (BD Biosciences). Following permeabilization, cells were stained with intracellular monoclonal antibodies 30–40 min at 4 °C, washed with wash buffer, acquired on the LSR II (BD Biosciences), and analyzed using FlowJo software. For intracellular cytokine staining of T cells infiltrating the CNS, mice were sacrificed and perfused with 20 ml of PBS. Spinal cord tissues were mechanically homogenized, resuspended in 30% Percoll, and slowly layered on top of 70% Percoll. After centrifugation at 1,300*g* for 20 min, the inflammatory cells infiltrating the CNS were retrieved from the 30/70% Percoll interface. Lymphocytes were prepared for intracellular flow cytometry staining as described above using the indicated antibodies.

### Luminex assay

Released cytokines expression was measured by fluorescent bead-based multiplex assay (Millipore) in accordance with the manufacturer's instructions. Samples were acquired with Luminex microplate reader and quantified using Upstate BeadView software.

### ELISPOT assay

Immobilon-P (PVDF) plates (Millipore, Bedford, MA, USA) were activated using 35% ethanol for 1 min and coated with 4 μg ml^−1^ of purified mouse anti-IL-17A and anti-IFN-γ (both from BD Biosciences, San Diego, CA, USA) overnight at 4 °C. Plates were blocked with 1% BSA, equal number of LN or spleen cells were loaded per well and incubated with increasing doses of MOG_35–55_ for 36 h at 37 °C. Plates were then washed and biotinylated anti-IFN-γ and anti-IL-17A (2 μg ml^−1^) were added and incubated overnight at 4 °C. Afterwards, the plates were again washed and incubated at room temperature for 2 h with alkaline phosphatase (Sigma, St Louis, MO, USA). Plates were then developed in BCIP/NBT (Sigma) solution. A computer-assisted ELISPOT Image Analyzer (Cellular Technology Limited, Cleveland, OH, USA) was used to count the spots.

### Proliferation assay

Cells were cultured in 96-well plate in triplicate at 2–5 × 10^5^ cells per well, and 200 μl per well was plated with different concentrations of MOG_35–55_ peptide. After 48 h of culture, 1 μCi 3H-thymidine (NEN Life Science Products, Boston, MA, USA) was added in 20 μl of media to each well for another 16–18 h. The CPM per well was counted by scintillation counter (Perkin-Elmer, Waltham, MA, USA). Data is presented as the mean CPM of triplicate wells per condition.

### Western blotting

Cells were lysed in RIPA buffer (25 mM Tris·HCl pH 7.6, 150 mM NaCl, 1% NP40, 1% sodium deoxycholate, 0.1% SDS, Thermo Scientific) used alongside protease and phosphatase inhibitor mixtures (Roche Diagnostics and Sigma-Aldrich, respectively). For separation by electrophoresis, 20 μg of total protein was loaded onto a SDS–PAGE gel according to standard protocols and then transferred on a PVDF membrane. The resulting membranes were then blocked for 1 h using 5% powder skim milk in TBS-Tween 20 and afterwards were incubated overnight at 4 °C with primary Abs including Tiam1 rabbit mAb (Millipore, ST1070, dilution 1/1,000) and Rac1 rabbit mAb (BD Biosciences, Cat. No. 610650, dilution 1/1,000. Membranes were striped and re-probed with mouse anti-β-actin antibody (Sigma-Aldrich, A3854, dilution 1/500,000) as a loading control. Blots were washed 3–5 times with TBS-Tween 20 and then incubated for 1 h using the appropriate HRP-conjugated secondary antibodies. Lastly, membranes were developed with the Immobilon Western Chemiluminescent HRP kit (Millipore, Billerica, MA, USA). Western blot images included in Fig. 1b have been cropped for presentation. Full size images of western blots are presented in Supplementary Fig. 1.

### Proximity ligation assay (PLA)

All antibodies used in this assay (rabbit anti-Tiam1 (Santa Cruz Biotechnology, sc-872), mouse anti-Rac1 (abcam, ab33186), mouse anti RORγ (BD Biosciences, Cat. No. 562663)) were antecedently rigorously tested with western blot and immunocytochemistry/immunofluorescence. The specificity of the antibodies was assessed by the time course of expression and the subcellular localization of the signal. PLA was performed according to the manufacturers protocol (Duolink In Situ Red Starter Kit Mouse/Rabbit, DUO92101, Sigma), with minor modifications. Briefly, naive CD4 T cells were subjected to Th17 differentiation conditions. Cells were harvested at 0, 6, 12, 24 and 48 h after the initiation of differentiation, fixed with 4% paraformaldehyde (PFA) in PBS for 10 min at room temperature. Subsequently the cells were washed three times with PBS (5 min each wash) and blocked with blocking buffer (PBS containing 8% horse serum, 3% bovine serum albumin and 0.3% Triton-X) for 30 min at room temperature. At this point cells were divided into two batches, on which two parallel PLA assays were performed: one for the detection of Tiam/Rac1 interaction and one for the detection of Tiam1/RORγ interaction. The primary antibodies (rabbit anti-Tiam1 with mouse anti-Rac1 for one of the batches, and rabbit anti-Tiam1 with mouse anti RORγ for the other batch) were applied on the cells in blocking buffer overnight at 4 °C on an orbital shaker. On the following day, cells were washed three times with PBS (5 min each wash) and incubated with the ‘+' and the ‘−' PLA probes diluted at 1:5 in the blocking buffer for 1 h at 37 °C with gentle shaking. Subsequently the cells were washed two times (5 min each) with 1 × Wash Buffer A, which was provided with the kit. The cells were then incubated with the ligase containing ligation solution for 30 min at 37 °C with gentle shaking. Next, the cells were washed twice (2 min each) with 1 × Wash Buffer A and then incubated with the polymerase containing amplification solution for 100 min at 37 °C with gentle shaking. Following the amplification, the cells were washed two times (10 min each) with 1 × Wash Buffer B followed a 1 min was with 0.01 × Wash Buffer B. Cells were subsequently resuspended in a DAPI (nuclear counterstain) containing mounting medium (supplied with the kit), mounted on a glass microscopy slide, covered with coverslip, sealed with nail polish and imaged as described below.

### Confocal imaging and image analysis

A Zeiss LSM710 confocal laser scanning microscope was used to acquire confocal images using the 405 and the 561 nm laser lines with the pinholes set to 1 Airy units for both channels. A 40 × oil immersion objective was used (Zeiss EC-PlanNEOFLUAR 40 × /1.30 Oil DiC M27) with a 10 × digital zoom available in the Zeiss Zen acquisition software. The dimensions of the acquired 8 bit images were X:256, Y:256 and Z:24 pixels, while the resolution was X:0.083 μm, Y:0.083 μm and Z:0.052 μm. The images were saved as.czi files and analyzed in FIJI (ImageJ2).

The ‘3D object counter' function of FIJI (ImageJ2) was used to determine the number of Tiam1/Rac1 and Tiam1/RORγ interactions per cells. Due to the more than 500-fold signal amplification properties of the PLA assay, each interaction of a single Tiam1 and Rac1 molecule (or Tiam1 and RORγ molecule) can be detected under the microscope as a fluorescent sphere. The nuclear counterstain (DAPI) was used for image segmentation when determining the subcellular localization (cytoplasmic versus nuclear) of the PLA signal. The Z stacks of 10 cells were analyzed per time point for both the Tiam1/Rac1 and the Tiam1/RORγ PLA assays.

### Subcellular fractionation and co-IP

Naïve CD4 T cells were differentiated to Th17 cells for 48 h (as described above) and subsequently fractionated using the NE-PER Nuclear and Cytoplasmic Extraction Kit (Thermo Scientific, Cat. No. 78833) according to the manufacturer's instructions. Briefly, 4–5 million differentiated CD4^+^ T cells were lysed in CER I, the lysate was vortexed, CER II was added to the lysate, vortexed again and let to sit on ice for 1 min. After centrifugation with 16,000*g* for 5 min the supernatant (the cytoplasmic fraction) was transferred to a clean tube. The pellet (containing the nuclei) was resuspended in ice-cold NER buffer, vortexed every 10 min for 40 min, and subsequently spun down with 16,000*g* for 10 min. The resulting supernatant (the nuclear fraction) was transferred to a pre-chilled microcentrifuge tube and the amounts were determined with the Pierce BCA Kit (Thermo Scientific, Cat. No. 23225) according to the manufacturer's instructions. For co-IP 100 μg of cytoplasmic protein and 100 μg of nuclear protein was used as input. As a pre-clearing step the different fractions were pre-incubated with 125 μl of Protein A Magnetic Beads (New England Biolabs, S1425S) for 1 h on a rotational mixer at 4 °C. Subsequently magnetic field was applied to the tubes, the supernatants transferred to clean tubes and the magnetic beads discarded. Each sample was incubated overnight with either 2 μg of rabbit anti-Tiam1 antibody (Santa Cruz Biotechnology, sc-872) or 2 μg of rabbit IgG in a final volume of 1 ml on a rotating mixer at 4 °C. Next day 125 μl of Protein A Magnetic Beads per sample was added to the cell lysates and incubated for 1 h on a rotational mixer at 4 °C. Subsequently, the magnetic beads were separated from the lysates with a magnet and washed three times with IP buffer (Thermo Scientific, Cat. No. 87788). Finally, the co-immunoprecipitated proteins were eluted from the beads with loading buffer (containing LDS Sample Buffer (Invitrogen, NP0007) and Sample Reducing Agent (Invitrogen, NP0009), loaded on a 4–12% Bis-Tris gel and separated by electrophoresis. The immunoprecipitated and co-immunoprecipitated proteins were detected by the following antibodies using the ECL detection method (Thermo Scientific, Cat. No. 32109): mouse anti RORγ (BD Biosciences, Cat. No. 562663, dilution 1/500), mouse anti-Rac1 (Abcam, ab33186, dilution 1/500), mouse anti lamin A/C (Cell Signaling, Cat. No. 4777, dilution 1/1,000) and goat anti paxillin (Santa Cruz Biotechnology, sc-7336, dilution 1/1,000). Immunoblot images included in Fig. 5g have been cropped for presentation. Full size images of western blots are presented in Supplementary Fig. 7.

### Expression analysis by real-time PCR

RNA from 50–100 × 10^3^ T cells was purified using Stratagene RNA kit and directly transferred into the RT reagent using the Applied Biosystems Taqman reverse transcriptase reagents. Samples were then subjected to real-time PCR analysis on the Applied Biosystems PRISM 7000 Sequencer Detection System (Applied Biosystems, Foster City, CA, USA) using standard conditions. Genes analyzed were detected with commercially available assays (Applied Biosystems). The relative mRNA abundance was normalized against GAPDH.

### ChIP-qPCR

Naive CD4^+^ CD44^low^CD62L^hi^ T cells were sorted by flow cell sorter and were polarized to Th17 phenotype for 48 h. ChIP was performed according to the protocol described in Bassil *et al*.^33^ Cell lysates were used for immunoprecipitation with anti-RORγt, anti-Tiam1, and anti-Rac1 (Santa Cruz Biotechnology: sc-28559, sc-872 and sc-217, respectively) and were compared with control IgG. For the CNS2 ChIP, the following primers were used for the detection of RORγt, Tiam1 and Rac1 binding: Fwd: ATGGGCCTCTCTTTCCACTGATG, Rev: GGAATTTGTGGTGGAAGGGAGTG according to previous report^36^.

### Luciferase reporter assay

PGL3 luciferase reporter plasmids encoding the *Il17* promoter with CNS2 and control plasmid were kindly provided by Dr Chen Dong^36^. Human embryonic kidney 293T cells were seeded in culture plates the day before transfection according the manufacturer's instructions. The cells confluency at the day of the transfection was 40–80%. 293T cells were transfected with luciferase reporter constructs together with the indicated plasmids using Effectene Transfection Reagent kit (Qiagen). Cells were cultured overnight then treated with Luciferase Assay System kit reagents (Promega), and results were acquired on Wallac 1420 Viktor2 plate reader (Perkin-Elmer).

### Histological analysis

Fourteen days after immunization, mice were sacrificed, and spinal cords were fixed in Bouin's fixative and then embedded in paraffin. Slides were processed for hematoxylin and eosin stains, and foci of inflammation were counted in a blinded fashion.

### Statistical analysis

The Mann–Whitney test was used for analysis of clinical disease. Statistical evaluations of luciferase activity, cytokine production and cell frequency measurements were performed using the unpaired Student's *t*-test. Values of *P*<0.05 were considered to be statistically significant.

### Data availability

Microarray data of CD4^+^ T cells from MS patients and healthy controls was downloaded from Gene Expression Omnibus (accession code GSE32988). The authors declare that the data that supporting the findings of this study are available within the article and its Supplementary Information Files.

## Additional information

**How to cite this article:** Kurdi, A. T. *et al*. Tiam1/Rac1 complex controls *Il17a* transcription and autoimmunity. *Nat. Commun.* **7**, 13048 doi: 10.1038/ncomms13048 (2016).

## Supplementary Material

Supplementary Information Supplementary figures 1-9
