# Supplementary material for: Tiam1/Rac1 complex controls Il17a transcription and autoimmunity
Source: Nat Commun. 2016 Oct 11;7:13048. doi: 10.1038/ncomms13048 (PMC5062600; doi:10.1038/ncomms13048)
Supplement: Supplementary Information — Supplementary figures 1-9 [file ncomms13048-s1.pdf]

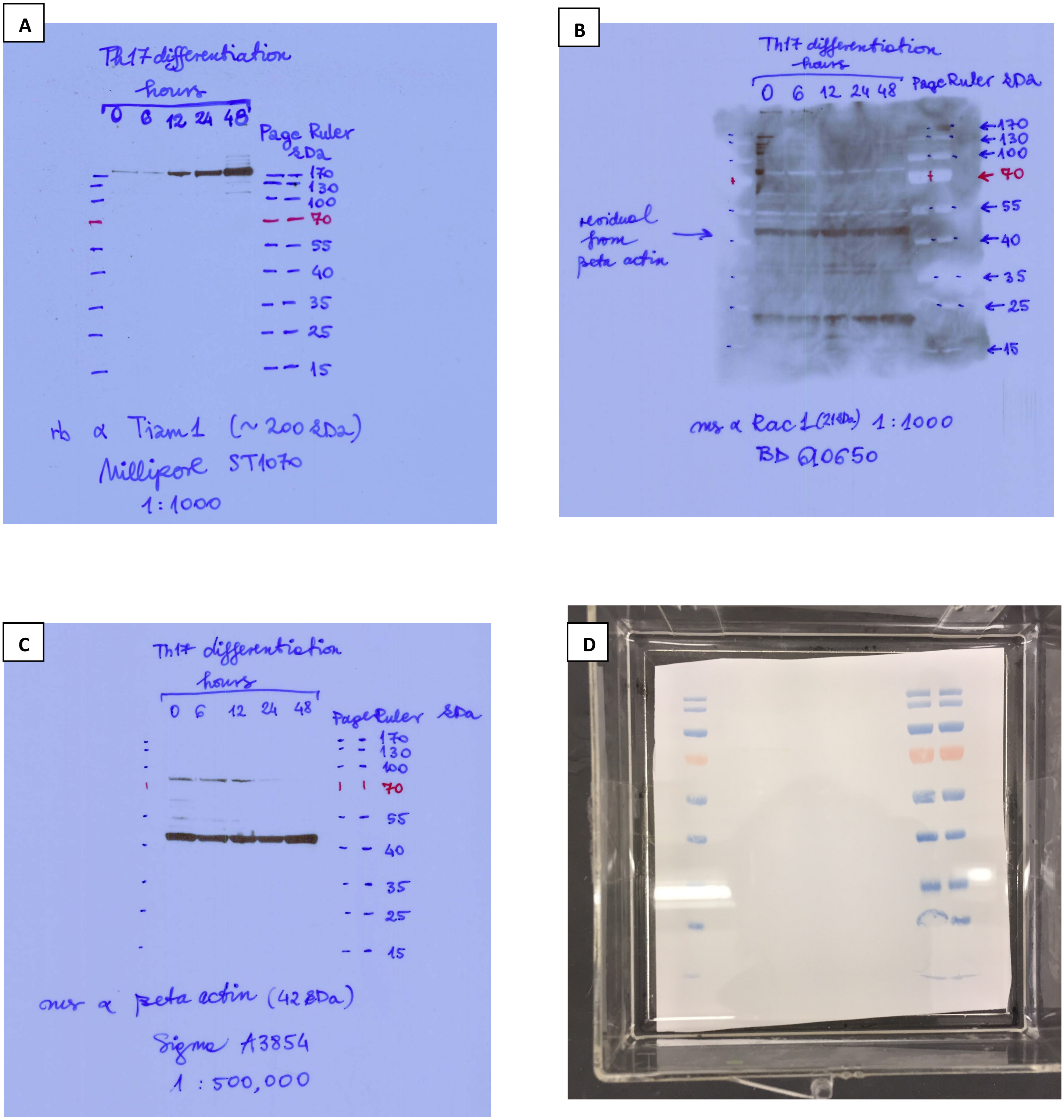

Supplementary Fig. 1. Full sized Western Blots corresponding to Tiam1 (A), Rac1 (B), and β-actin blots (C) along with Western Blot membrane (D) from Figure 1B with ladder indicating molecular weight values.

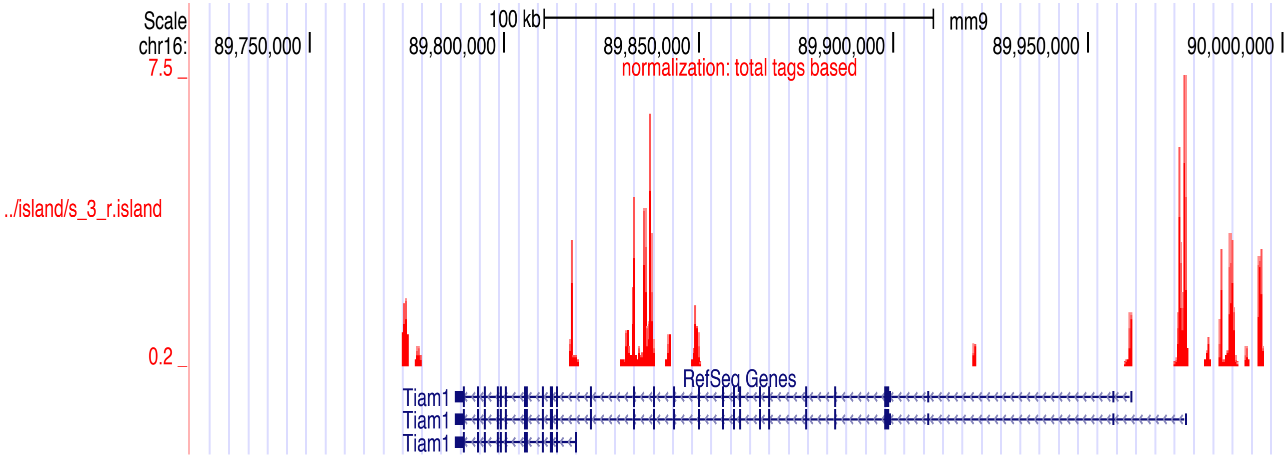

Supplementary Fig. 2. STAT3 Genomic Binding Sites at the Tiam1 Locus.

The STAT3 ChIP-seq data was extracted from GEO (GSM652877) and submitted to USCS genome browser (Mus musculus, assembly: mm9) for visualization as a custom track. Light blue arrowheads show the orientation of the gene, thick lines represent the exons and the thin blue lines represent the introns. In red, the STAT3 ChIP-seq peaks can be seen in the promoter region upstream of transcription start site (TSS) of both the short and the long Tiam1 isoforms. Chromosome number (chr16) and sequence position are also indicated on the figure.

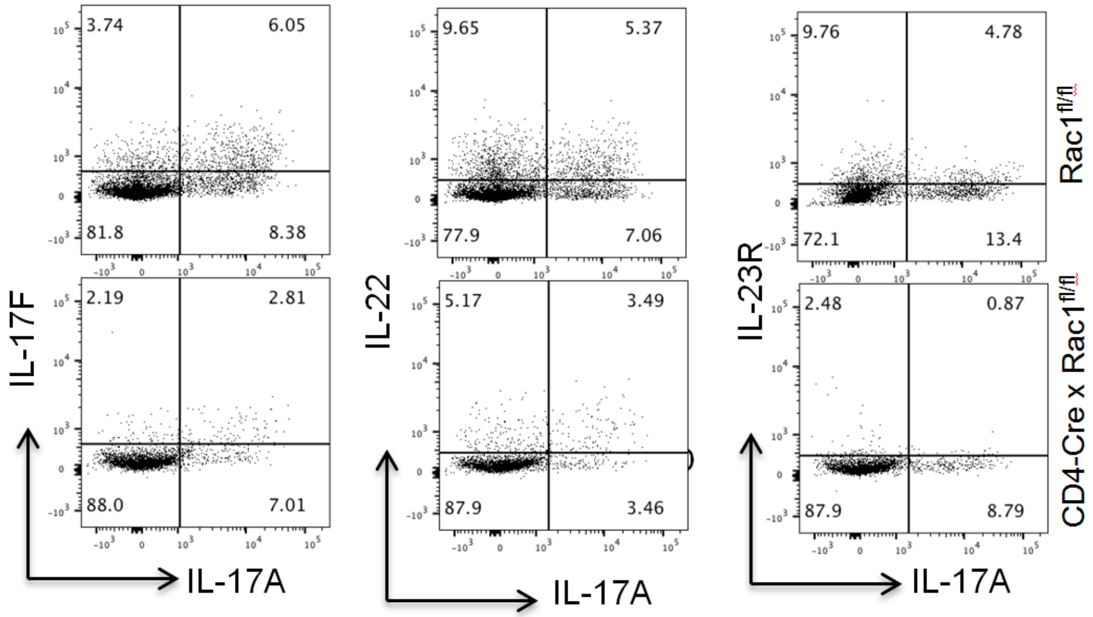

Supplementary Fig. 3. Rac1 is required for Th17 cytokine production *in vitro.*

Cytokine expression by flow cytometry. Naive CD4$^+$ T cells were isolated from *CD4-Cre x Rac1*$^{fl/fl}$ and control *Rac1*$^{fl/fl}$ mice and naïve CD4$^+$ T cells were stimulated *in vitro* under pathogenic Th17 cell condition with IL-1β, IL-6 and IL-23 for 4 days. Lymphocytes were activated with PMA/ionomycin for 4 hours followed by intracellular flow cytometry staining. Numbers in quadrants indicate percent of positive cells. Data shown are representative of two independent experiments.

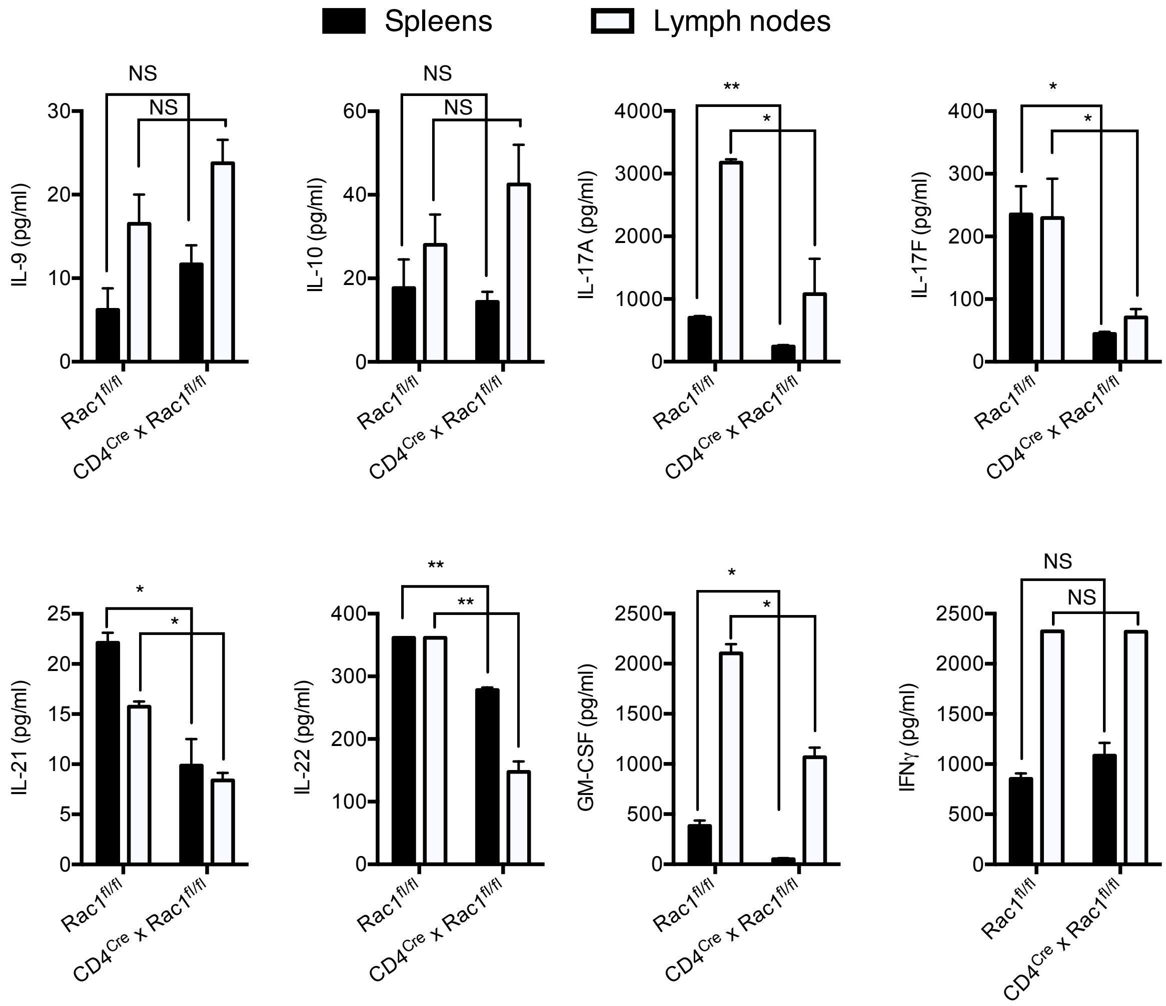

Supplementary Fig. 4. Rac1 is required for in vivo Th17 cytokine production.

Cytokine profile of $MOG_{35\text{-}55}$-reactive T cells in immunized Rac1 conditional knockout mice. *CD4*^{cre} *x Rac1*$^{fl/fl}$ and control *Rac1*$^{fl/fl}$ mice were immunized with $MOG_{35\text{-}55}$ in CFA and spleen and lymph node cells were isolated 10 days after immunization and cells were re-challenged with $MOG_{35\text{-}55}$ peptide *in vitro* for 36 hours. Supernatants were collected and cytokine expression was measured by Luminex. Data shown (mean ± s.e.m.) are representative of three independent experiments.

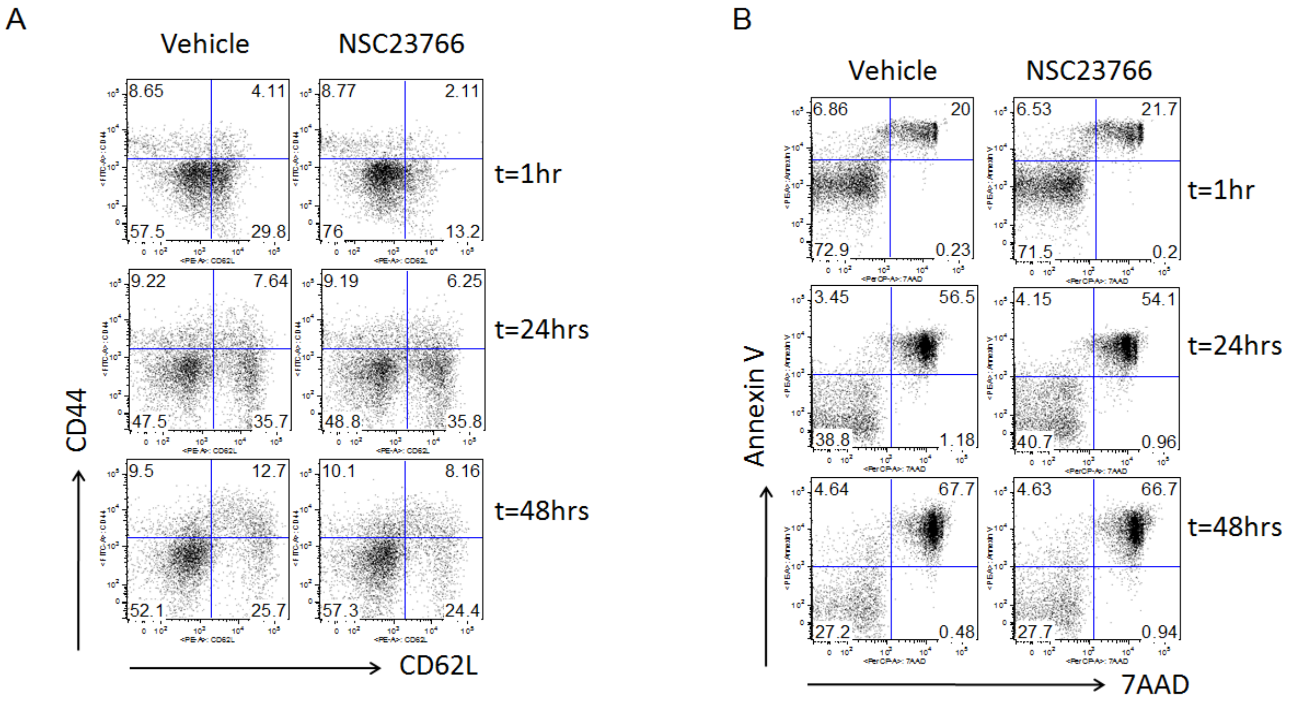

Supplementary Fig. 5. NSC23766 Does Not Affect $CD4^+$ T cell Activation or Survival. Naive $CD4^+$ T cells were isolated from WT mice and were stimulated *in vitro* with anti-CD3/CD28 (1 mg/ml) in the presence of NSC23766 (94 mM) or control solvent. A, CD4+ T cell activation was measured using antibodies against CD44 and CD62L surface molecules 1h, 24h and 48h after treatment. B, Similarly, cell survival was assessed at the above mentioned time points using AnnexinV and 7AAD staining. Numbers in quadrants indicate percent of positive cells.

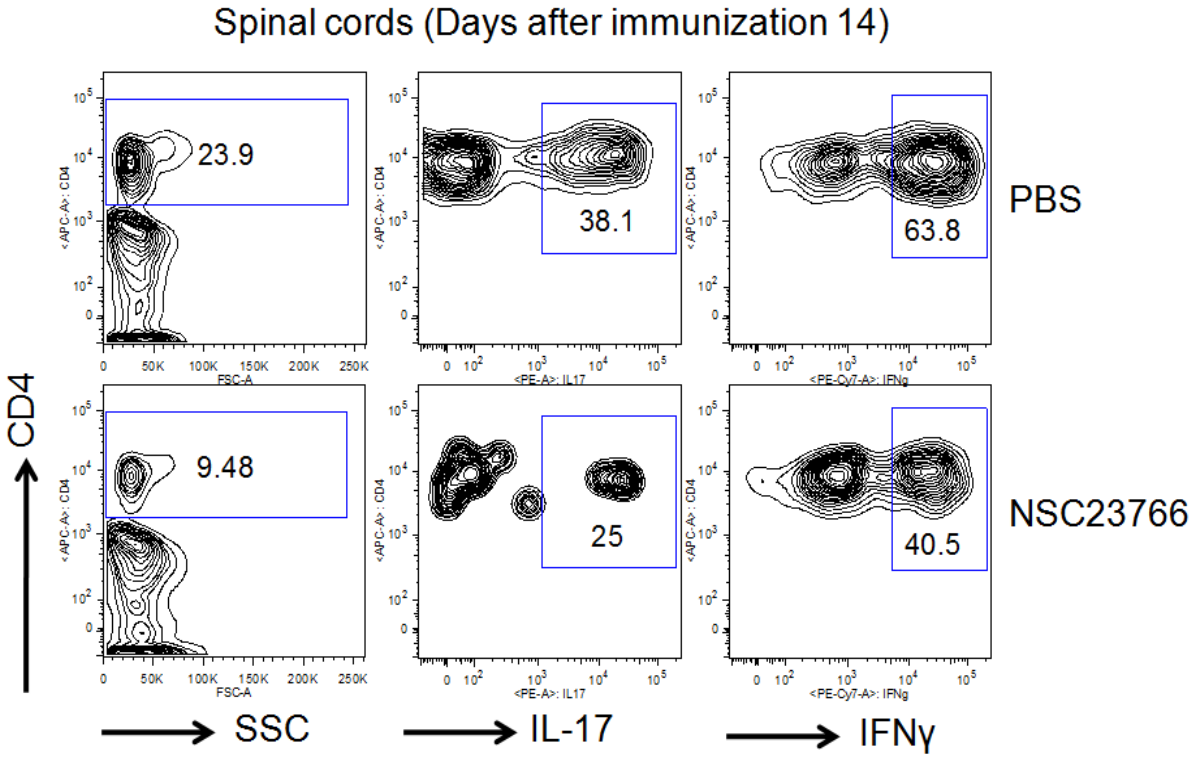

Supplementary Fig. 6. $CD4^+$ T cell Infiltration in EAE Mice Following NSC23766 Treatment. C57BL/6 WT mice were immunized with 100 µg $MOG_{35–55}$/CFA and received eight doses i.p. of NSC23766 (8×5 $mg.kg^{-1}$) every day starting from the day of immunization. Control mice received PBS treatment according to the same regimen. On day 14 after immunization (peak of the disease), mice were sacrificed and perfused with PBS. Spinal cord tissues were digested with collagenase IV (Sigma-Aldrich) for 30 min at 37°C, resuspended in 30% Percoll, and loaded onto 70% Percoll. After centrifuge at 1300 × *g* for 20 min, the CNS inflammatory cells were retrieved from the 30/70% Percoll interface. Lymphocytes were activated with PMA/ionomycin followed by intracellular flow cytometry staining. Frequency of infiltrated $CD4^+$ T cells is shown. Numbers in quadrants indicate percent of positive cells.

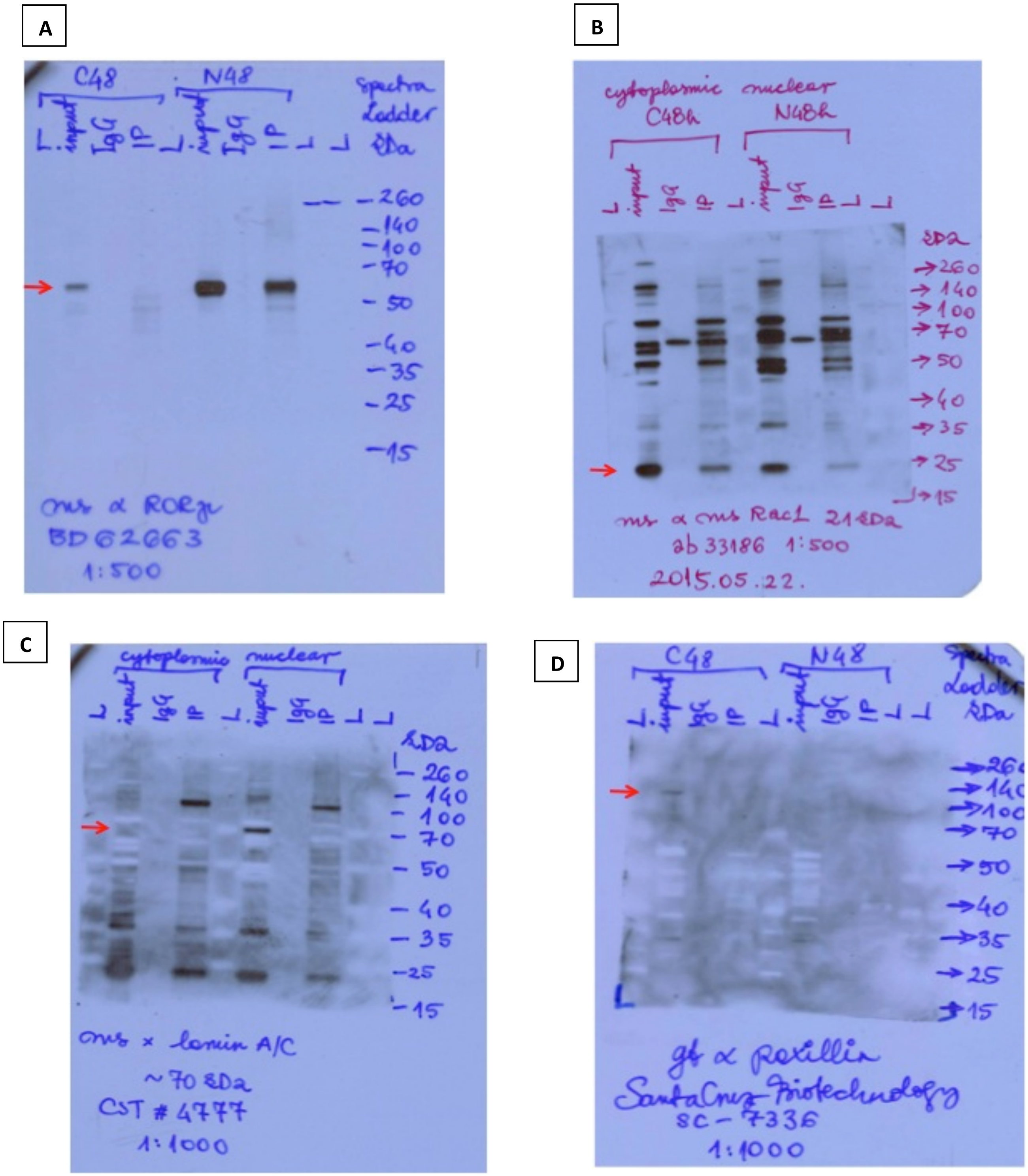

Supplementary Fig. 7. Full sized co-immunoprecipitation blots corresponding to RORγt (A), Rac1(B), lamin (C), paxillin (D) presented in Figure 5G with ladder indicating molecular weight values.

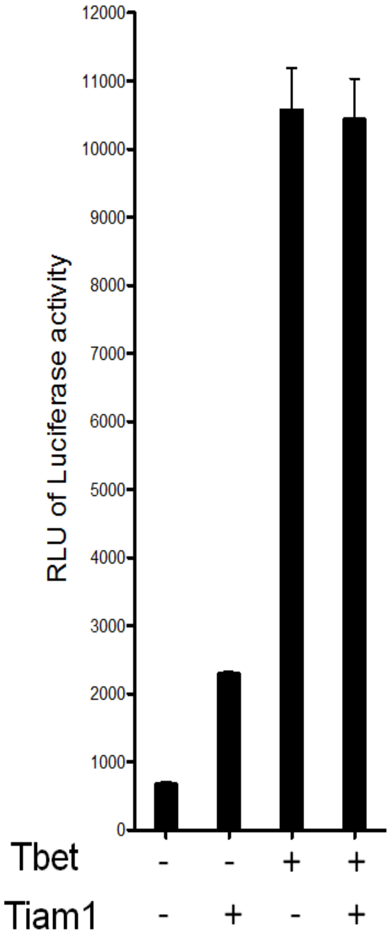

Supplementary Fig. 8. Tiam1 has no Effects on T-bet-Mediated *Ifng* Promoter Activation. HEK293T cells were transfected with a constant amount of pGL3-Ifng vector in the presence of the indicated constructs, cells were cultured for 48 hours, cell protein extracts were prepared, and luminescence was measured. Data represent mean ± s.e.m of a representative experiment each performed in triplicate.

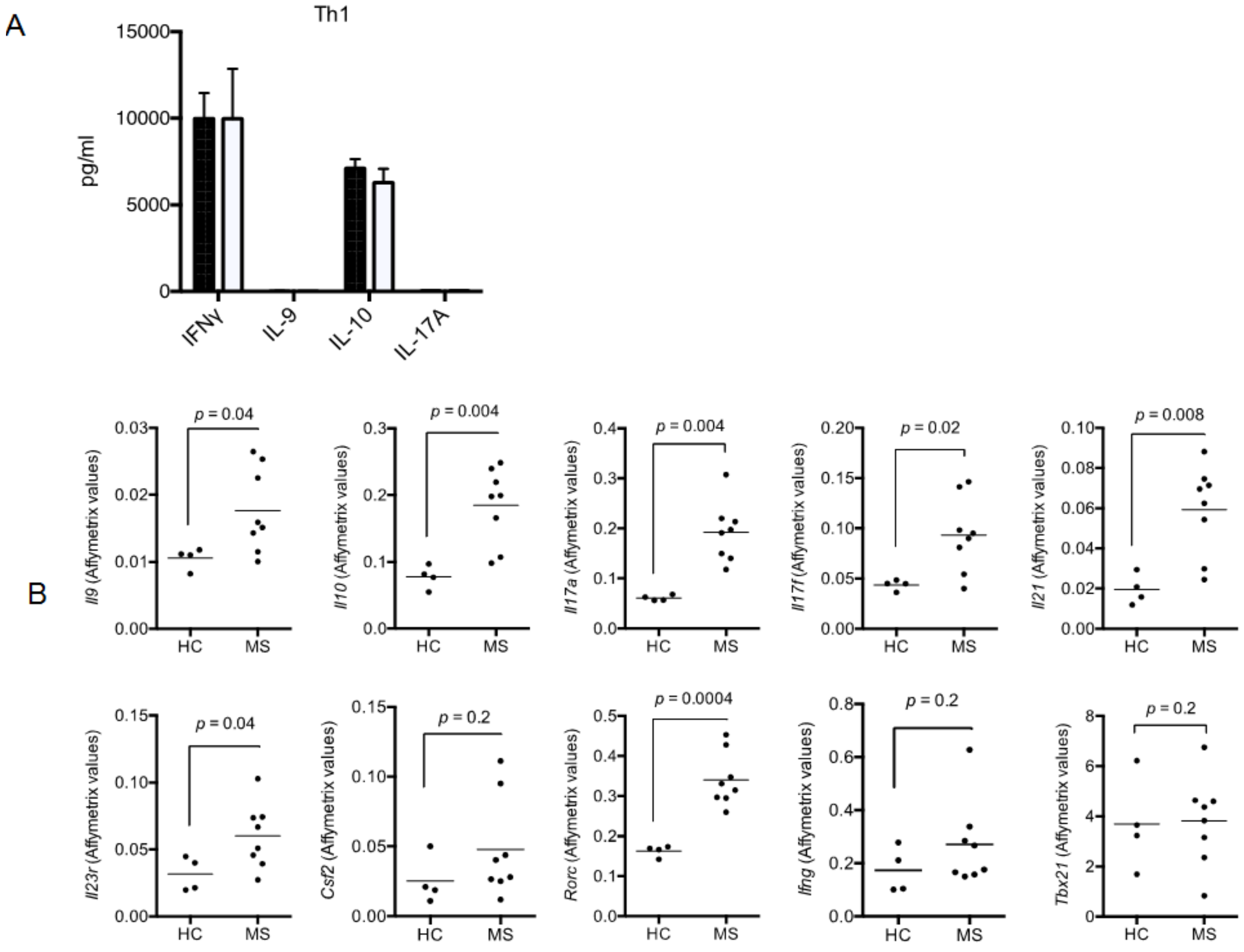

Supplementary Fig. 9. Cytokine profile of CD4+ T cells in multiple sclerosis (MS) patients and healthy controls (HC). (**A**) Cytokine profile of NSC23766-treated Th1 cells by Luminex. Supernatants were collected from human Th1 cell cultures at the end of the differentiation (7 days) and were analyzed by bead-based Luminex assay according to the manufacturer's instructions. Data represent mean ± s.e.m. of a representative experiment each performed in triplicate. (**B**) Cytokine expression profiling of $CD4^+$ T cells from MS patients (n=8) and healthy controls (n=4) was analyzed by Affymetrix gene array and deposited on Gene Expression Omnibus (GEO) (GSE32988). Affymetrix values are show in the y-axis. Each dot represents an individual. Statistical significance defined by P <0.05 by student *t* test.
